# Distinct lipid profile, low-level inflammation, and increased antioxidant defense signature in HIV-1 elite control status

**DOI:** 10.1016/j.isci.2021.102111

**Published:** 2021-01-28

**Authors:** Maike Sperk, Flora Mikaeloff, Sara Svensson-Akusjärvi, Shuba Krishnan, Sivasankaran Munusamy Ponnan, Anoop T. Ambikan, Piotr Nowak, Anders Sönnerborg, Ujjwal Neogi

**Affiliations:** 1 Division of Clinical Microbiology, Department of Laboratory Medicine, Karolinska Institute, ANA Futura, Campus Flemingsberg, Stockholm 14152, Sweden; 2 Centre for Infectious Disease Research, Indian Institute of Science (IISc), CV Raman Avenue, Bangalore, Karnataka 560012, India; 3 Department of Medicine Huddinge, Division of Infectious Diseases, Karolinska Institute, I73, Karolinska University Hospital, Huddinge, Stockholm 141 86, Sweden; 4 Department of Microbiology and Immunology, University of Missouri, Columbia, MO 65211, USA

**Keywords:** Immunology, Proteomics, Metabolomics

## Abstract

HIV-1 elite controllers (EC) are a rare but heterogeneous group of HIV-1-infected individuals who can suppress viral replication in the absence of antiretroviral therapy. The mechanisms of how EC achieve undetectable viral loads remain unclear. This study aimed to investigate host plasma metabolomics and targeted plasma proteomics in a Swedish HIV-1 cohort including EC and treatment-naïve viremic progressors (VP) as well as HIV-negative individuals (HC) to get insights into EC phenotype. Metabolites belonging to antioxidant defense had higher levels in EC relative to VP, whereas inflammation markers were increased in VP compared with EC. Only four plasma proteins (CCL4, CCL7, CCL20, and NOS3) were increased in EC compared with HC, and CCL20/CCR6 axis can play an essential role in EC status. Our study suggests that low-level inflammation and oxidative stress at physiological levels could be important factors contributing to elite control phenotype.

## Highlights

• Increased acylcholine as unique HIV-1 positive elite controllers (EC) feature
• Physiological oxidative stress and inflammation profile in EC
• Increased in CCL4, CCL7, CCL20, and NOS3 in EC compared with HIV-ve control
• CCR6-CCL20-dependent anti-HIV mechanism can play an essential role in EC status

## Graphical abstract

**Figure:**
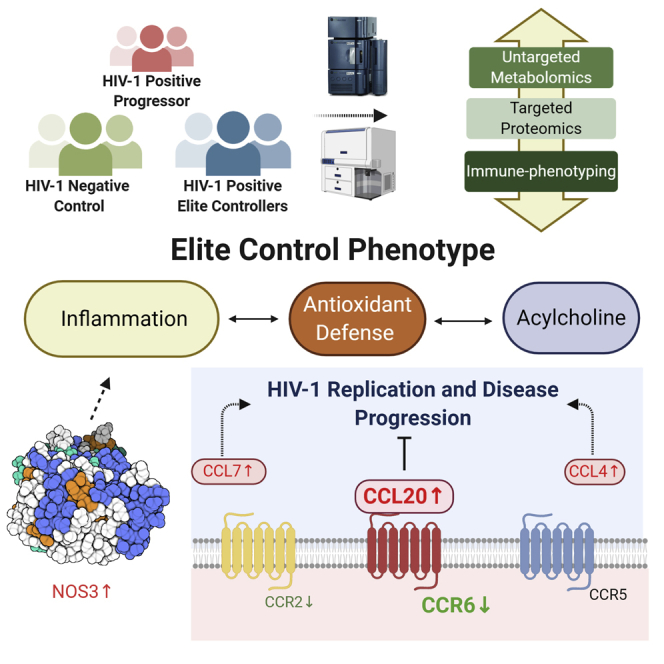

## Introduction

HIV-1 elite controllers (EC) are a heterogeneous group among HIV-1-infected individuals who can suppress viremia in the absence of antiretroviral therapy (ART). Although specific definitions for EC vary, EC maintain low levels of HIV-RNA and physiological levels of CD4^+^ T cell counts without showing any clinical symptoms of HIV-1 infection and progression to AIDS for a prolonged period. They are a rare patient subset (<1% of HIV-1-infected individuals) but of great interest in HIV-1 research because they might hold a key for developing HIV-1 cures or vaccines (Olson et al., 2014). It is, however, unclear which mechanisms lead to viral suppression in EC.

It is thought that the major part of viral control can be attributed to host factors rather than viral factors (e.g., infection with a defective or attenuated viral strain). Several small studies have indicated that host genetic factors such as human leukocyte antigen (HLA), for example, HLA-B∗57:01, HLA -B∗27:05, HLA-B∗52, or HLA-A∗25, and CCR5 Δ32, can play protective roles in HIV-1 infection (Gonzalo-Gil et al., 2017), but larger multi-cohort studies failed to prove this hypothesis. Our earlier proteo-transcriptomic study reported that multiple immune pathways can play a synergistic role in controlling the viral replication in EC and that this group is heterogeneous with distinct properties (Zhang et al., 2018).

The progressive development of high-throughput technologies makes it possible to collect and analyze large amounts of genetic and molecular data simultaneously. In this study, we aimed to characterize the metabolic signature of the elite control status in HIV-1-positive individuals and elucidate the mechanism by integrating targeted proteomics and immunological assays from a well-defined EC cohort. We observed EC phenotype-specific lipid profiles linked with antioxidant defense and inflammation; together, they might be important factors contributing to the control of viral replication.

## Results

### General findings

**Figure 1:**
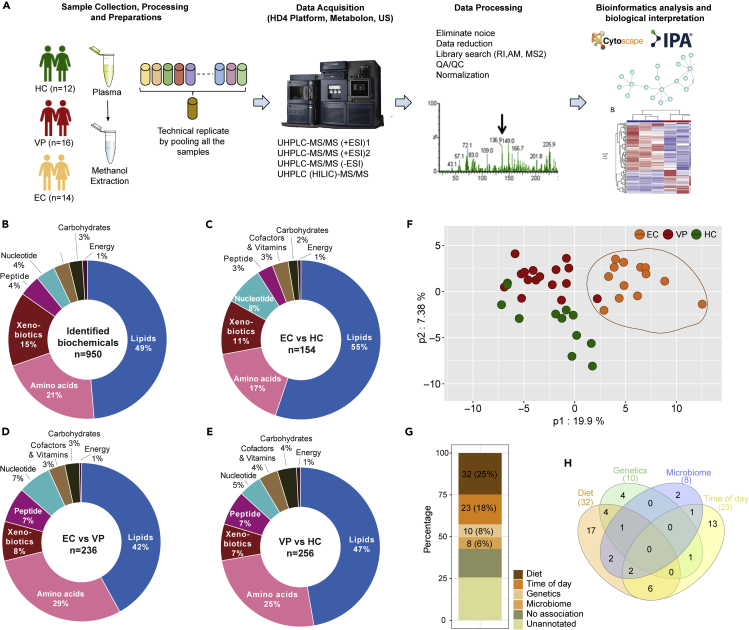
Study design and general findings (A) Study design. Plasma samples of 12 HIV-negative individuals (HC), 16 HIV-1-infected treatment-naïve individuals with viremic progression (VP), and 14 HIV-1 Elite controllers (EC) were prepared and analyzed by ultra-high-performance liquid chromatography-tandem mass spectrometry (UHLC-MS/MS). Raw data processing, biochemical identification, quality control, and data normalization were performed according to Metabolon pipeline. Results were analyzed by appropriate bioinformatical and statistical methods *in house*. (B–E) Doughnut chart with proportions of each super pathway (B) based on the total number of detected and identified metabolites in all groups (950 metabolites) (C) based on the number of metabolites with significantly different levels in EC and HC (p < 0.05, 154 metabolites) (D) based on the number of metabolites with statistically significantly different levels in EC and VP (p < 0.05, 236 metabolites) based on the number of metabolites with statistically significantly different levels in VP and HC (p < 0.05, 256 metabolites). See also Figure S2. (F) Partial least squares discriminant analysis (PLS-DA) of the 129 EC-specific signature metabolites revealing distinct clustering of EC from HC and VP. Data shown include samples of HC (n = 12), EC (n = 13), and VP (n = 16). (G) EC-specific signature metabolites mapping reported metabolites that are associated with diet, microbiome, genetics, lifestyle, and time of sampling (Bar et al., 2020). (H) Venn diagram showing the overlap of the identified metabolites that are related to diet, microbiome, genetics, lifestyle, and time of sampling.

We used three age-, gender-, and body mass index (BMI)-matched cohorts, EC (n = 14), viremic progressor (n = 16; VP), and HIV-negative controls (n = 12; HC) and performed plasma metabolomics analysis using four different ultra-high-performance liquid chromatography and mass spectrometry (UHPLC-MS/MS) methods (Figure 1A). A total of 950 biochemicals were identified. The majority of them belonged to the class of lipids (49%), followed by amino acids (21%) (Figure 1B). Group-wise comparisons of detected metabolites and percentage of each biochemical class involved is shown in Figures 1C–1E. In the unsupervised principal component analysis (PCA), all samples, regardless of study group, clustered together except for one EC sample (EC06), which was separated from the others (Figure S1). This sample EC06 was statistically classified as an outlier caused by technical errors and was thus excluded from further analyses of the metabolomics data. For the remaining 41 samples (EC with n = 13, HC with n = 12, VP with n = 16), a group effect was seen for 294 of the detected biochemicals, and similar amounts of altered biochemicals were found when comparing EC versus VP (236) and VP versus HC (256). We observed that biochemicals were often decreased in VP compared with the other groups.: Of the molecules that were altered compared with EC 86.7% were reduced in VP and of those that were changed compared with HC 87.3% were lower in VP. This indicates a strong metabolic differentiation of VP from the other two groups. The metabolites differing between EC and VP as well as of HC and VP is depicted in volcano plots (Figures S2A and S2B). EC and HC showed only a modest separation (Figure S2C). Next, we identified an EC-specific signature by taking the union between the comparisons of EC versus VP and EC versus HC and subtracting VP versus HC metabolites and identified that 129 metabolites were EC specific. Using these 129 metabolites, all EC clustered together segregating from VP and HC in partial least squares-discriminant analysis (PLS-DA) (Figure 1F). Although our population was matched with general diet, we do not have extensive diet data. Therefore, we mapped the metabolites that were earlier reported to be associated with diet, microbiome, genetics, lifestyle, or time of the sampling in the day in a population-based studies (Bar et al., 2020), which identified that 43% (56/129) of the metabolites were not associated or not annotated with any of the factors reported earlier and thus, as of today, not significantly associated with lifestyle (Figure 1G). The Venn diagram shows that most of the metabolites are associated with more than one factor, with 17 metabolites associated with diet only (Figure 1H).

### Components involved in lipid metabolism contribute most to group segregation

**Figure 2:**
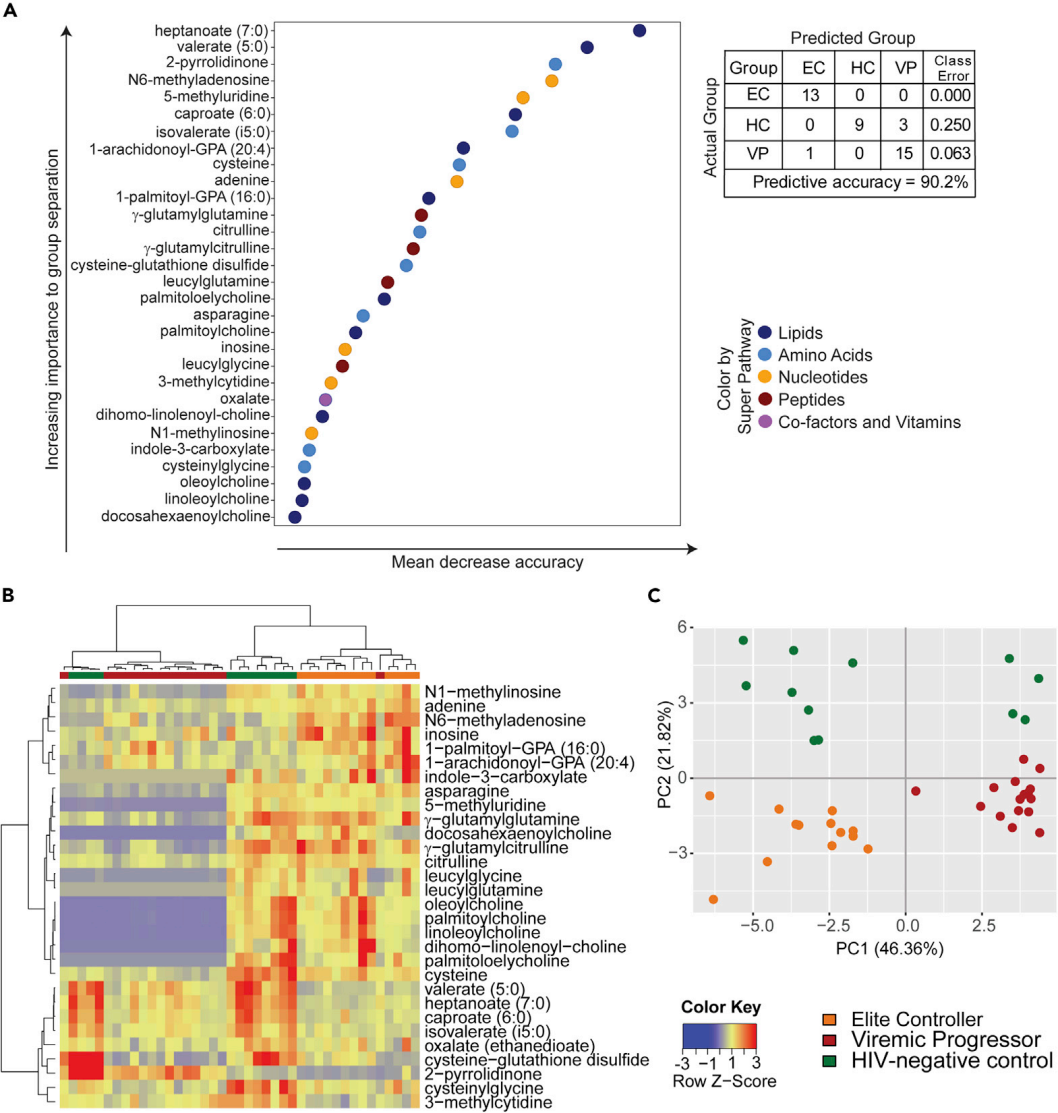
Random forest and supervised analyses of metabolites (A) Random forest (RF) analyses showing the top 30 metabolites that contribute to group separation and the super pathways the metabolites belong to (see color-coded legend). Biochemicals involved in lipid and amino acid metabolism dominate the top-ranked intermediates. The table represents predictive accuracy, predicted, and actual grouping of the samples. See also Figure S3 for RF analyses between two study groups. (B) Supervised hierarchical clustering analyses based on the top 30 metabolites using the Pearson method with Ward algorithm. (C) Supervised principal component analyses (PCA) based on the top 30 metabolites. Data shown includes samples of HC (n = 12), EC (n = 13), and VP (n = 16).

To rank the biochemicals according to how important they are for group separation, we used random forest (RF). The top 30 biochemicals revealed by this method are presented and subsumed under their biochemical class (Figure 2A). A predictive accuracy above 50% implies hereby that results are not random chance. When comparing all three groups, the high predictive accuracy of 90.2% was obtained with biochemicals involved in lipid and amino acid metabolism (11 and 8 out of 30 metabolites, respectively) dominating the top-ranked intermediates followed by nucleotide metabolism (6 out of 30 metabolites, Figure 2A). An even higher predictive accuracy was given for VP versus EC samples (96.6%), whereas 85.7% was seen for VP versus HC and 100% for EC versus HC (Figure S3). For all of them, key differences were suggested in lipid and amino acid metabolism.

Supervised clustering analyses were used for the top 30 ranked metabolites that were revealed by RF. Samples of the EC and VP group dispersed moderately demonstrating a low metabolic variation within these groups. We observed the segregation of EC and VP with only one VP sample clustering within EC. The clustering was consistent in hierarchical clustering analysis (HCA) (Figure 2B) as well as in PCA (Figure 2C) and indicated a robust metabolic differentiation between EC and VP. HC samples were in between those of EC and VP showing a modest separation of EC and HC (Figures 2B and 2C). However, four of the twelve HC samples clustered together with VP implying some differences within this sample group. As already seen in volcano plots belonging to unsupervised analyses, supervised HCA also revealed a decrease of metabolites in VP compared with EC and HC (more than two-thirds of the top 30 metabolites, colored blue in the heatmap, Figure 2B).

### Unique acylcholines profile in EC

**Figure 3:**
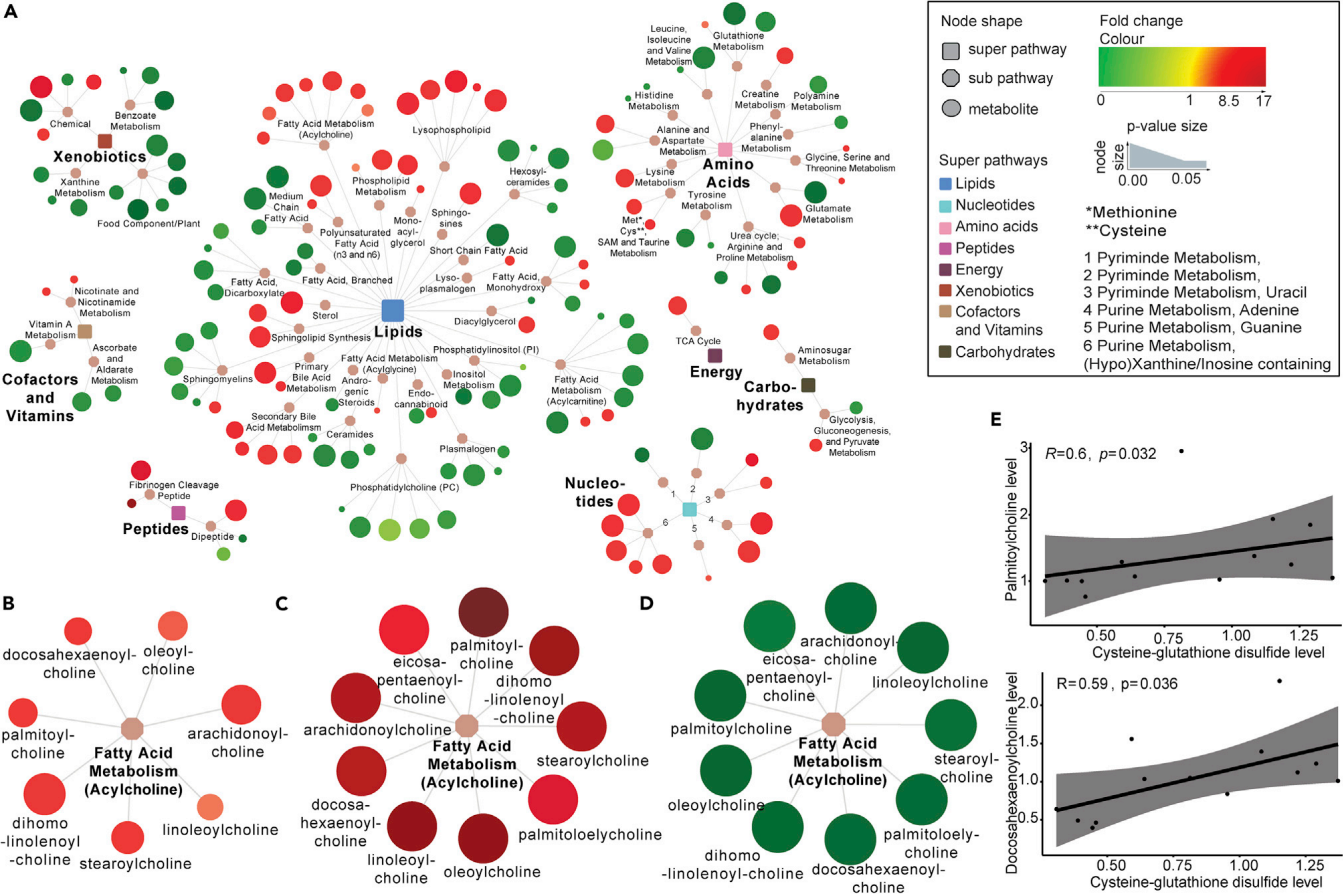
Unique acylcholines profile in EC (A) Network analyses of the metabolites that were significantly different in EC versus HC (154 metabolites). Rectangular shapes represent the eight super-pathways that are shown in different colors according to legend. Octagonal shapes are used for subpathways belonging to the eight super pathways. Circular shapes show the single metabolites, where red indicates increased levels and green indicates decreased levels in EC relative to HC. The size of the circles indicates p value: the bigger the size, the lower the p value. Lines connect each metabolite to its respective subpathway and subpathway to their respective super-pathways. See also Figure S4 for network analyses between EC and VP as well as HC and VP. (B–E) (B–D) Network showing the sub pathway *Fatty acid metabolism acylcholines* and metabolites belonging to it, with red indicating increased levels and green indicating decreased levels. Size of the circles indicated p value of (B) EC versus HC, (C) EC versus VP, and (D) VP versus HC. (E) Spearman correlation analyses between metabolites were performed for EC samples and revealed a moderate correlation between levels of cysteine-glutathione disulfide and palmitoylcholine (R = 0.6, p value = 0.032) as well as levels of cysteine-glutathione disulfide and docosahexaenoylcholine (R = 0.59, p = 0.036). Respective scatterplots are presented. Data shown include samples of HC (n = 12), EC (n = 13), and VP (n = 16).

Pathway mapping of all detected biochemicals reflects the decrease of metabolites in VP compared with EC and HC (Figure S4). Because most metabolites detected belong to lipids, it is not surprising that most differences between groups were seen for these biomolecules. Concordant with the general findings, VP had lower lipid levels compared with EC and HC, including several metabolites belonging to polyunsaturated fatty acids, lysophospholipids, phospholipid metabolism, or lysoplasmalogen. In contrast, diacylglycerol levels were highest in VP and unchanged in EC compared with HC. No one-sided trend was observed between EC and HC though, not in the metabolic network as a whole and neither for lipids (Figure 3A). Instead, some lipid subclasses (e.g. lysophospholipids, phospholipid metabolism, as well as primary and secondary bile acid metabolism) were increased, and others (e.g. acylcarnitines, sphingomyelins, phosphatidylcholines, and ceramides) were decreased in EC. An increase or decrease was usually consistent within one subpathway (Figure 3A).

We further observed that lipids being part of acylcholines were reduced in VP and elevated in EC compared with HC. Every acylcholine detected was significantly and markedly decreased in VP subjects relative to HC. Interestingly, acylcholines were not decreased; instead, most of them were even increased in EC relative to HC, leading to markedly elevated acylcholines in EC relative to VP (3.5 < fold change< 17.5, Figures 3B–3D). Of note, choline levels were also elevated in EC compared with HC (p = 0.044) and VP (p < 0.001).

Interestingly, levels of 2 acylcholines (palmitoylcholine and docosahexaenoylcholine) correlated with the level of cysteine-glutathione disulfide that is involved in antioxidant defense (R = 0.6 and p = 0.032 for palmitoylcholine and R = 0.59 and p = 0.036 for docosahexaenoylcholine) (Figure 3E).

### Increased antioxidant defense in EC relative to VP linked to glutathione and one-carbon metabolism

**Figure 4:**
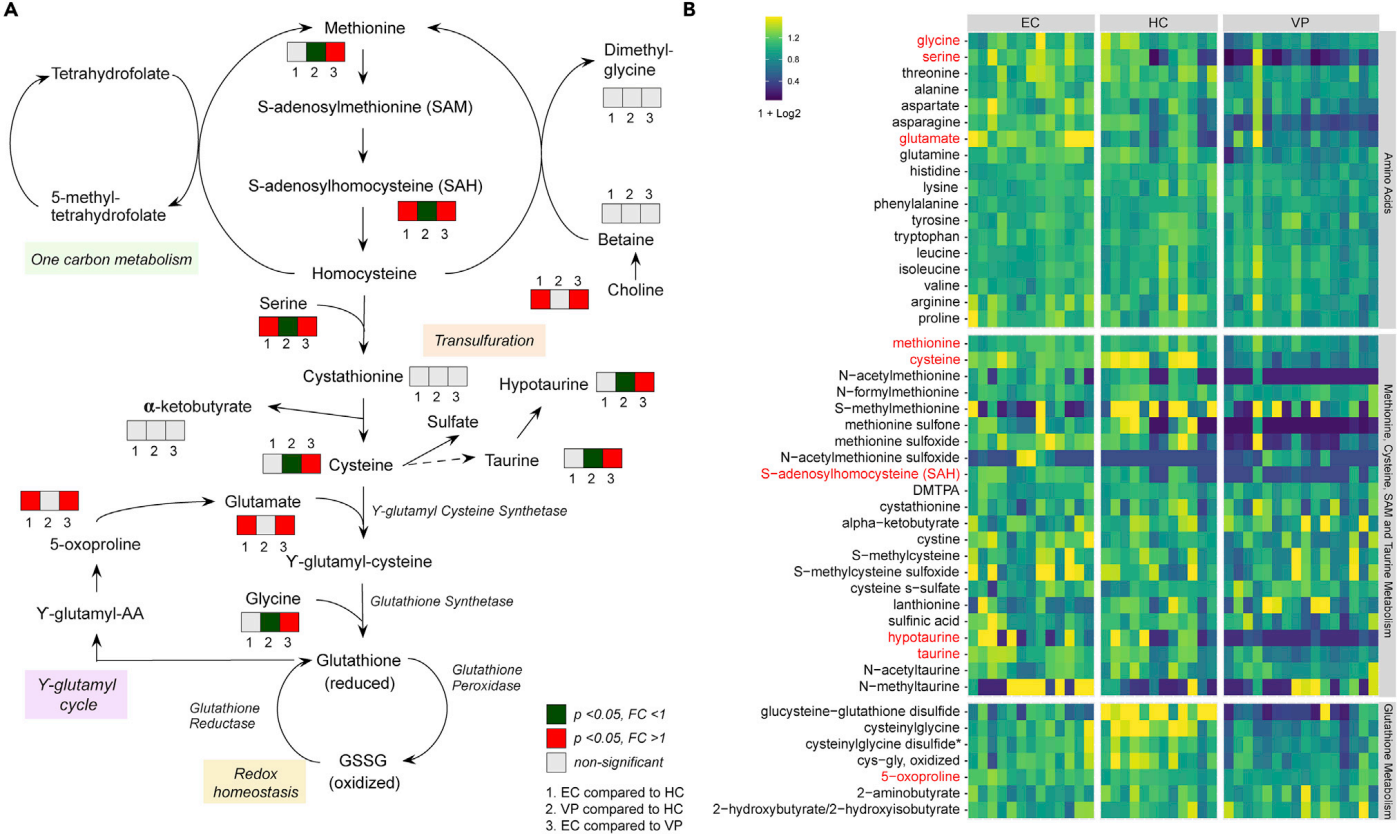
Increased antioxidant defense in EC relative to VP linked to glutathione and one-carbon metabolism (A) A schematic presentation of methionine metabolism that is linked to transsulfuration pathway, one-carbon metabolism, and glutathione metabolism. Methionine is transformed into S-adenosylmethionine (SAM) and S-adenosylhomocysteine (SAH) and finally converted into homocysteine, which is also connected to one-carbon metabolism. In the transsulfuration pathway, cysteine and homocysteine are interconverted through the intermediate homocysteine. Cysteine can give rise to the antioxidants taurine and hypotaurine and it is also part of *de novo* glutathione synthesis. As glutamate is one of the three peptides glutathione (GSH) constitutes of, there is a link to the gamma-glutamyl cycle. By reducing reactive oxygen species (ROS), GSH is transformed to its oxidized form GSSG and GSSG can be reverted to reduced GSH by the enzyme glutathione reductase. Boxes in neighborhood of a metabolite indicate that the respective metabolite was detected and quantified in all our samples. Box 1 shows comparison between EC and HC, box 2 between VP and HC, and box 3 between EC and VP. A gray box presents non-significant difference in plasma levels, red presents fold-change greater than one (with p value<0.05), and green fold-change smaller than one (with p value<0.05). (B) Heatmap representing levels of metabolites that are part of amino acids; methionine, cysteine, SAM, and taurine metabolism; and glutathione metabolism. Samples are grouped according to study group (EC, HC, VP). Metabolites written in red are part of the scheme shown under (A). Color depictures increasing log2 levels from blue via green to yellow. Data shown include samples of HC (n = 12), EC (n = 13), and VP (n = 16). See also Figure S5 and Table S1 for differences in respective metabolite levels between study groups.

As the choline levels may be linked to changes in oxidative stress and/or antioxidant defense, next we investigated glutathione metabolism linked with transsulfuration pathway and one-carbon metabolism, as the cellular tripeptide glutathione is a key cellular antioxidant compound regulating redox homeostasis (Figure 4A). Relative to VP, EC showed an increase in the methylation reaction product S-adenosylhomocysteine (SAH) (p < 0.001); the glutathione precursors cysteine (p < 0.001) and glycine (p = 0.004); the cysteine-derived antioxidants hypotaurine (p < 0.001) and taurine (p < 0.001); as well as the glutathione cycle intermediates cysteinylglycine (p = 0.004), 5-oxoproline (p < 0.001), glutamate (p < 0.001), and several gamma-glutamyl amino acids (Figures 4A and S5, and Table S1). Together, these data suggest that EC exhibited increased activity of the transsulfuration pathway toward *de novo* glutathione synthesis in addition to increased glutathione recycling compared with VP. In addition, methionine, which is linked to glutathione synthesis via the transsulfuration pathway, was also increased in EC compared with VP (p = 0.003), suggesting the aforementioned increases in glutathione synthesis in EC may also reflect elevated methionine availability. Furthermore, EC showed, relative to VP, increases in many oxidized intermediates including cysteine-glutathione disulfide (p = 0.012), cysteinylglycine disulfide (p = 0.002), oxidized cysteinylglycine (p = 0.04), cystine (p = 0.007), methionine sulfoxide (p < 0.001), and methionine sulfone (p < 0.001) (Supplementary S5a). Looking at EC and HC, significant increase was observed in 5-oxoproline (p < 0.046), glutamate (p < 0.001), serine (p = 0.048), and SAH (p = 0.02) in EC (Figures 4A and S5). The level of metabolites that are part of amino acids; methionine, cysteine, S-adenosylmethionine (SAM), and taurine metabolism, and glutathione metabolism and that are linked to abovementioned one-carbon metabolism, transsulfuration, gamma-glutamyl cycle, and redox homeostasis is presented in Figure 4B. Although increases in oxidized intermediates could reflect elevated oxidative stress, in the context of elevated glutathione synthesis/recycling and cysteine availability, this signature likely also reflects improved antioxidant defense mechanisms and greater detoxification of reactive oxygen species (ROS) in EC. Our data thus indicate that EC and HC have similar oxidative stress levels and antioxidant defense. This is further supported by the measurement of ROS in blood cells (Figure S6A) and subsequent gene expression analysis of some of the NRF2-antioxidant response element (ARE) signaling pathway genes: nuclear erythroid 2-related factor (*NRF2)*, NAD(P)H quinone dehydrogenase 1 (*NQO1)*, heme oxygenase 1 (*HO-1*), and Kelch-like ECH-associated protein 1 (*KEAP1*) (Figure S6B) where we do not see any statistical significance between EC (n = 14) and HC (n = 8) samples.

### Inflammation markers in EC relative to HC

**Figure 5:**
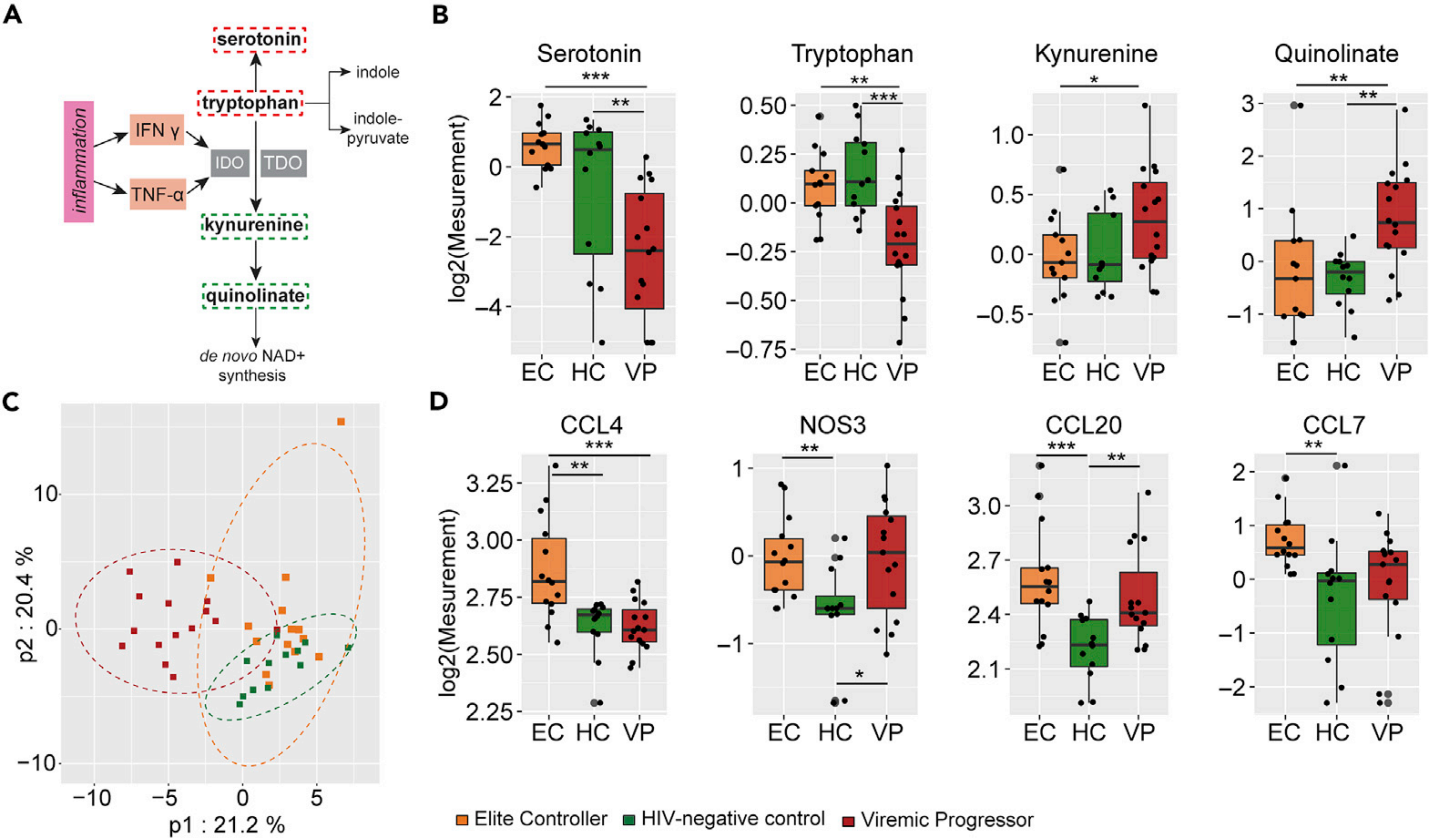
Inflammation markers (A) Tryptophan can be metabolized along several distinct pathways either giving rise to the neurotransmitter serotonin, along the kynurenine pathway that has been associated with inflammation and disease, or to several indole compounds. Kynurenine in liver is generated by tryptophan dioxygenase (TDO) but in extra-hepatic tissues by the enzyme indoleamine 2,3-dioxygenase (IDO), which is well characterized to be induced by the inflammatory cytokines, interferon-gamma (IFNγ), and tumor necrosis factor alpha (TNF-α). Kynurenine can further be metabolized to quinolinate that is a precursor of nicotinamide adenine dinucleotide (NAD^+^). Metabolites that were detected in samples are marked with rectangular broken lines around them; red indicates higher levels and green represents lower levels in EC relative to VP. IDO, Indoleamine 2,3-dioxygenase; IFN γ, Interferon gamma; NAD^+^, nicotinamide adenine dinucleotide; TDO, tryptophan 2,3-dioxygenase; TNF-α, tumor necrosis factor alpha. (B) Boxplots of metabolites that belong to kynurenine pathway. Log2 of serotonin, tryptophan, kynurenine, and quinolinate are presented for EC (orange), HC (green), and VP (red). Median values and interquartile ranges are indicated by bars. p values are determined by Mann-Whitney U test. ∗p value<0.05, ∗∗p value<0.01, and ∗∗∗p value<0.001. (C) Partial least squares-discriminant analysis (PLS-DA) including 41 proteins analyzed in plasma samples showing clustering of the three study groups EC (orange), HC (green), and VP (red) samples where VP samples cluster separately from HC and EC, whereas HC and EC intermingle to a small extend. (D) Boxplots of proteins that had significantly different levels in HC and EC (p < 0.05) revealed by Mann-Whitney U test. Log2 of CCL4, CCL7, CCL20, and NOS3 are presented for EC (orange), HC (green), and VP (red). Median values and interquartile ranges are indicated by bars. ∗p value<0.05, ∗∗p value<0.01, ∗∗∗p value<0.001. Data shown include samples of HC (n = 12), EC (n = 14), and VP (n = 16). See also Figure S6 for plasma levels of CRP and Neopterin.

As any kind of infection is often accompanied by systemic inflammation, and there is a known link between oxidative stress and inflammation, we were next interested in levels of inflammation markers. We hypothesized that improved antioxidant defense allows EC to keep the inflammation level low despite an earlier study suggesting increased inflammation in EC compared with HC (Li et al., 2015). On a metabolic level, we noticed lower levels of kynurenine and quinolinate together with increased levels of serotonin and tryptophan in EC compared with VP but no differences between EC and HC. This signature likely reflects an increased metabolism of tryptophan to kynurenine along kynurenine pathway in VP (but not in EC), which is associated with inflammation (Hopper et al., 2012) (Figures 5A and 5B). To further support this we used plasma proteomics markers of inflammation in HC and EC and compared levels of selected plasma proteins using two different approaches: re-analysis of data that was obtained by proximity extension assay (PEA) applying Olink immuno-oncology panel, which has partly been published earlier (Zhang et al., 2018), together with enzyme-linked immunosorbent assay (ELISA) for detection of C-reactive protein (CRP) and neopterin. In the proteomics data analyses, EC06 was included (EC with n = 14, HC and VP as for metabolomics). Out of 92 plasma proteins analyzed by PEA, 81 passed quality control and were used for statistical analyses. PLS-DA showed group clustering with VP being separated from the other two groups, whereas HC and EC intermingled to some extent (Figure 5C). This clustering is similar to that of the metabolomics data. Out of the 81 proteins, four were statistically different in EC and HC: CCL4 (p = 0.001), CCL7 (p = 0.002), CCL20 (p < 0.001), and nitric oxide synthase 3 (NOS3, p = 0.006) (Figure 5D). All of them were increased in EC compared with HC. ELISA confirmed elevated CCL20 levels in EC relative to HC (data not shown). Median (IQR) CRP levels in the study groups indicate a trend of elevated CRP plasma levels in EC (1.32 [0.95–2.49]) compared with HC (1.07 [0.69–1.71]) but lower than in VP (2.38 [2.25–3.66]). That was, however, statistically not significant (EC versus HC p = 0.2469 and EC versus VP p = 0.1469) (Figure S7A). Neopterin plasma levels were increased in VP but not in EC compared with HC (Figure S7B). These data indicate that EC has a rather similar inflammation profile to HC and different from VP.

### Decreased CCR6 surface expression in EC

**Figure 6:**
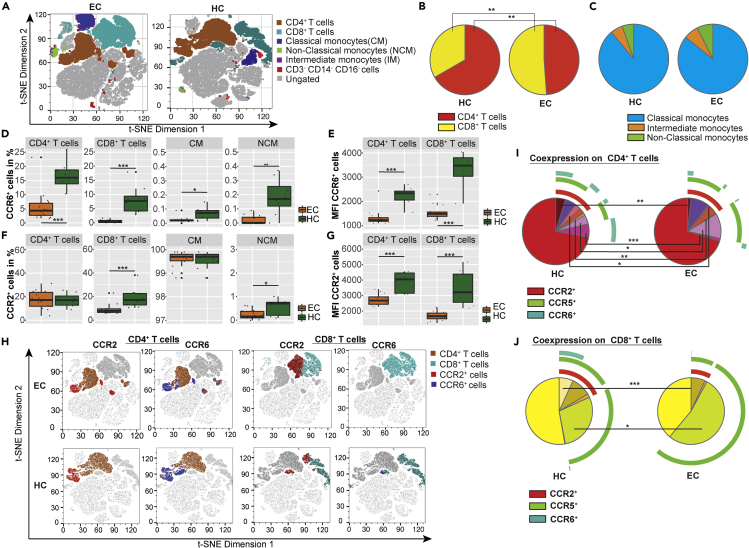
Flow cytometry analyses (A) Differences between EC (left) and HC (right) in overall clustering of PBMCs acquired by flow cytometry. Viable singlet cells of each sample were downsampled to 10,000, and individual downsampled samples of EC and HC, respectively were concatenated. t-SNE analysis was performed with 2,000 iterations with perplexity of 20 and learning rate of 1,000. The two plots show t-SNE dimension 1 and t-SNE dimension 2 based on expression of CD4, CD8, CD14, CD16, CCR2, CCR3, CCR5, and CCR6. CD4^+^ T cells are shown in brown, CD8^+^ T cells in light blue, classical monocytes in dark blue, non-classical monocytes in green, intermediate monocytes in purple, cells that are triple-negative for CD3, CD14, and CD16 in red; and ungated cells in gray. (B) Pie charts depicture different proportions of CD4^+^ (yellow) and CD8^+^ (red) T cells in HC (left) and EC (right). (C) No difference is observed in the proportion of classical (blue), intermediate (orange), and non-classical (green) monocytes between HC (left) and EC (right). Proportions are presented in pie charts. (D–G) Boxplots represent expression frequency of median fluorescence intensity (MFI) of selected surface receptors on different cell populations, revealed by flow cytometry analysis for EC (orange) and HC (green). Median values and interquartile ranges are indicated by bars. p values are determined by Mann-Whitney U test. ∗p value<0.05, ∗∗p value<0.01, and ∗∗∗p value<0.001. (D) Expression frequency of CCR6 on CD4^+^ T cells and CD8^+^ T cells as well as on classical and non-classical monocytes. (E) MFI of CCR6 on CD4^+^ T cells and CD8^+^ T cells. (F) Expression frequency of CCR2 on CD4^+^ T cells and CD8^+^ T cells as well as on classical and non-classical monocytes. (G) MFI of CCR2 on CD4^+^ T cells and CD8^+^ T cells. (H) Differences between EC (upper row) and HC (lower row) in surface expression of CCR2 and CCR6 on CD4^+^ and CD8^+^ T cells. t-SNE analysis of downsampled and concatenated samples was performed as described under (A) Presented t-SNE plots show t-SNE dimension 1 and t-SNE dimension 2. CD4^+^ T cells are highlighted in brown in the two columns on the left and CD8^+^ T cells are shown in light blue in the two columns in the right. Lymphocytes expressing CCR2 are colored in red and those expressing CCR6 in dark blue. (I and J) Pie charts depicture the co-expression of CCR2, CCR5, and CCR6 on lymphocytes for HC (left) and EC (right). The expression of CCR3 is very low and has therefore been neglected in these illustrations. (I) Co-expression on CD4^+^ T cells. (J) Co-expression on CD8^+^ T cells. Data shown include samples of HC (n = 8) and EC (n = 14). See also Figure S7 for additional flow cytometry data and Figure S8 for the gating strategy.

**Figure 7:**
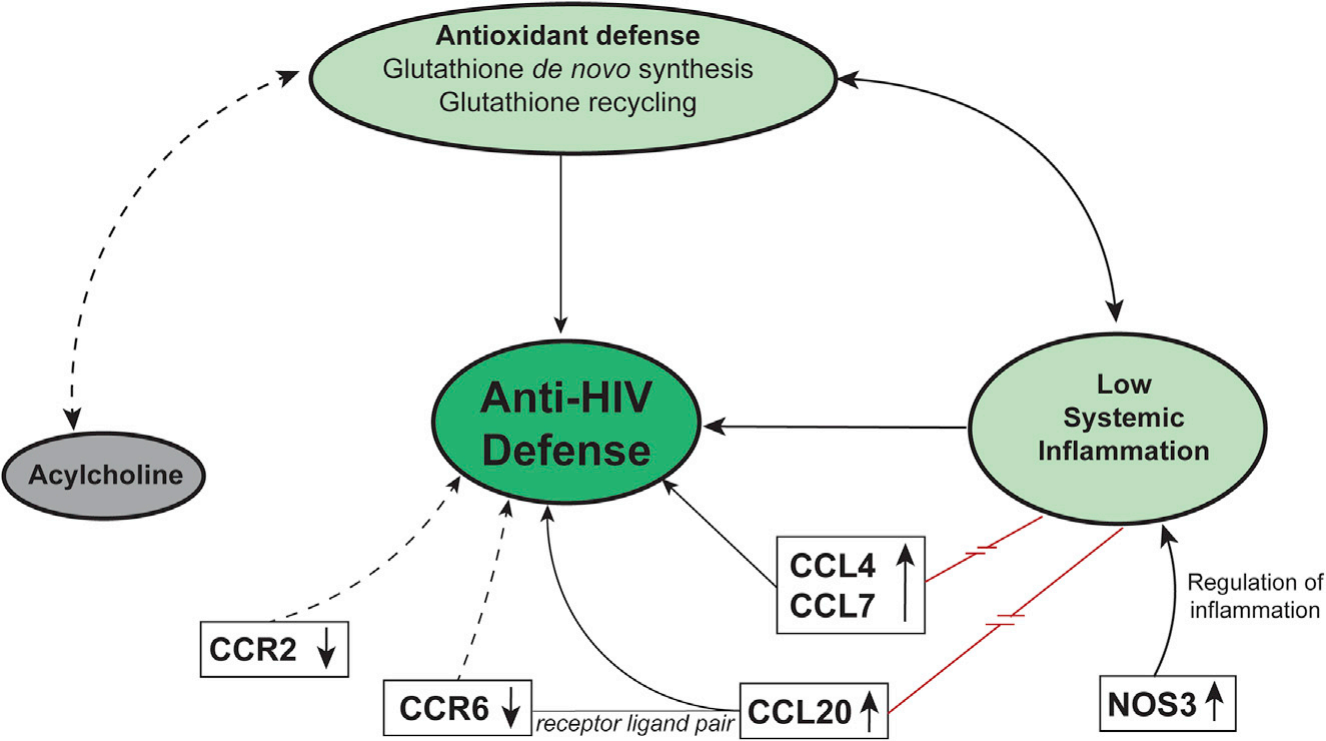
The proposed hypothesis of viral control mechanisms in EC A summary of our findings that might contribute to viral control. EC showed a physiological state of antioxidant defense and low systemic inflammation except for increased plasma levels of CCL4, CCL7, CCL20, and NOS3. Chemokine receptors CCR2 and CCR6 were downregulated on lymphocytes in EC. The acylcholine levels in plasma samples of EC were increased. Arrows depicture known relations between features. The red line illustrates conflicting findings. Broken lines with arrows represent hypothesized relations between features that need to be investigated in future studies.

Cytokine-mediated signaling occurs through the binding and subsequent activation of cytokines to their specific receptors. Alterations in either cytokine levels, in expression profiles of their specific receptors, or in both could indicate changes in cytokine signaling pathways. Due to elevated plasma levels of the cytokines CCL4, CCL7, and CCL20 in EC relative to HC, we were interested in the surface expression of their respective receptors, CCR2 (ligand CCL7), CCR3 (ligand CCL7), CCR5 (ligand CCL7 and CCL4), and CCR6 (ligand CCL20), on peripheral blood mononuclear cells (PBMCs). Flow cytometry analyses were therefore performed on a subpopulation of gender-matched EC (n = 14) and HC (n = 8) samples of the study cohort. Antibody panel was chosen to discriminate CD4^+^ T cells (CD3^+^CD4^+^), CD8^+^ T cells (CD3^+^CD8^+^), and monocytes expressing a classical (CD14^+^CD16^-^), non-classical (CD14^−^CD16^+^), and intermediate (CD14^+^CD16^+^) phenotype. EC had significantly decreased numbers of CD4^+^ T cells and significantly increased counts of CD8^+^ T cells compared with HC (Figures 6A and 6B). No differences were seen in the proportion of monocytes (Figures 6A and 6C). To our surprise, percentages of cells expressing CCL20 receptor CCR6 were reduced in EC in all cell subpopulations (Figure 6D) except for intermediate monocytes (Figure S8A). For CD4^+^ and CD8^+^ T cells the reduction was substantial, whereas only 0.07% of classical monocytes and 0.17% of non-classical monocytes were CCR6^+^ in HC making the difference to EC marginal (CCR6 expression in 0.02% of classical and <0.01% of non-classical monocytes). Furthermore, surface expression levels of CCR6 were reduced on CCR6 expressing CD4^+^ and CD8^+^ T cells in EC compared to HC (Figure 6E). Small differences were seen regarding frequency and distribution of CCR2 expression (Figure 6F). Frequencies of CCR2 expressing cells were reduced in CD8^+^ T cells (7.81% versus 17.00%) and in non-classical monocytes (0.16% versus 0.72%) in EC, whereas CCR2 surface expression levels were decreased on CD4^+^ and CD8^+^ T cells in EC compared with HC (Figure 6G). Different distribution of CCR2 and CCR6 expression on CD4^+^ and CD8^+^ T cell populations are depicted in t-SNE plots (Figure 6H). The frequency of CCR3-expressing cells was generally very low in all cell populations, with an upregulation in CD4^+^ T cells (0.58% versus 0.29%) and downregulation in non-classical monocytes (<0.01% versus 0.13%) in EC (Figure S8A). The frequency of cells expressing CCR5, which is receptor for CCL7 and CCL4, and also a co-receptor for HIV-1 entry, was similar in EC and HC in all examined cell populations (Figure S8A). Although a trend was seen for higher frequencies of CCR5-expressing CD8^+^ T cells in EC, that reached not statistical significance (p = 0.068) (Figure S8A). No differences were seen for any of the receptors in intermediate monocytes (Figure S8A). Analysis of co-expression of several receptors revealed significant differences between EC and HC in both CD4^+^ (Figure 6I) and CD8^+^ (Figure 6K) T cell subsets that co-express CCR2, CCR5, and CCR6 with lower frequencies in EC compared with HC. Further, amount of CD4^+^ T cells co-expressing CCR5 and CCR6 as well as CCR2 and CCR6 is reduced in EC relative to HC. The clustering of different cell subsets and expression of surface markers is also depicted in t-SNE plots (Figure S8B). The gating strategy is given in Figure S9. These data indicate that CCR6/CCL20 chemokine axis along with CCR2/CCR5/CCL4/CCL7 signaling, which plays a role in HIV-1 entry and in antiviral immunity (Aebersold and Mann, 2003; Lee and Körner, 2017), are modulated in EC. A summary of important findings in our EC cohort is presented in Figure 7.

## Discussion

Our study indicated a distinct plasma metabolic signature with physiological oxidative stress and an HC-like inflammation profile as well as an increased antioxidant defense compared with viremic progressors as a hallmark of EC phenotype. The levels of acylcholines were markedly increased compared with HC and VP, making it a unique EC-specific feature. These characteristics might be important factors contributing to EC phenotype. Moreover, distinct cytokine-mediated signaling attributed to CCR6-CCL20-dependent mechanism and CCR2 can play an essential role in antiviral immunity, but their specific role in EC remains to be clarified.

Lipid abnormalities have been reported in HIV-1-infected individuals as a result of infection (but also as an effect of ART). By altering intracellular pathways involved in cellular metabolism in general or lipid signaling in particular, an optimal environment can be created for viral replication (Jain et al., 2018). Despite heterogeneous, the acylcholines showed marked increases in all the EC. The biological role of acylcholines is not well understood. Chemically, acylcholines are esters formed from choline and a carboxylic acid, and the acylcholines we detected mainly derived from long-chain fatty acids (Hastings et al., 2016). Moreover, a recent study indicated that levels of most of the acylcholines were not influenced by diet or the microbiome in the general populations (Bar et al., 2020). Thus, the fatty acids and their salts respectively or choline itself might have a biological function in EC status. One of the acylcholines detected, namely arachidonoylcholine, is known as a cholinergic agonist that binds to and activates cholinergic receptors (Hastings et al., 2016). Whether arachidonoylcholine or other acylcholines (besides the classical agonist acetylcholine) and acylcholine receptor signaling play a role in EC viral control needs further investigation. Furthermore, a protective effect of arachidonoylcholine, oleoylcholine, linoleoylcholine, and docosahexanoylcholine against H_2_O_2_-induced cytotoxicity has been discussed (Akimov et al., 2020).

Interestingly, acylcholine levels in EC also correlated with levels of metabolites involved in glutathione metabolism. Methionine is a key molecule involved in the regulation of several metabolic processes, such as sulfur metabolism and redox homeostasis. During a process called Met oxidation, the sulfur residues in methionine can also be oxidized by ROS into methionine sulfoxide or methionine sulfone. Since levels of methionine, its two oxidation products, as well as other oxidized intermediates were higher in EC compared with VP, one could suspect elevated oxidative stress in EC. Methionine is, however, also the start of the methionine-cycle-generating homocysteine that is further used in the transsulfuration pathway. Finally, this pathway leads to *de novo* glutathione synthesis from methionine to regulate redox homeostasis (Figure 4A) (Lee and Gladyshev, 2011). In our study, certain metabolites that are part of methionine cycle (SAH) or glutathione recycling (cysteine, glycine, cys-gly, 5-oxoproline) were increased in EC compared with VP, suggesting that EC exhibit greater antioxidant defense activity than VP. Taurine, which derives from cysteine via the intermediate hypotaurine, has also been reported to have antioxidant properties (Ripps and Shen, 2012). Both hypotaurine and taurine levels were higher in EC compared with VP. HIV-1 infection is associated with increased ROS production and oxidative stress combined with glutathione deficiency and suppression of other antioxidant pathways (Ivanov et al., 2016). In our EC cohort though, levels of biochemicals that are part of antioxidant defense pathways were similar to the ones in HC cohort. SAH and 5-oxoproline, both used for *de novo* glutathione synthesis, had even higher levels in EC compared with HC. Cysteinylglycine was the only metabolite of the glutathione metabolism with significantly lower levels in EC compared with HC but still higher compared with VP. This signature could reflect that EC, but not VP, are able to keep redox homeostasis at levels seen in HIV-uninfected individuals. Our finding is consistent with a recent study, where spontaneous loss of virologic control and transition from EC status was associated with increased oxidative stress and deregulated mitochondrial function prior to loss of control. Hence, oxidative stress could also be a potential determinant of EC status (Tarancon-Diez et al., 2019). Oxidative stress might be beneficial for HIV-1 replication, and glutathione deficiency is associated with impaired survival in HIV-1 infection. In contrast, glutathione treatment prevents infection of new cells through impaired virion budding and release *ex vivo* (Herzenberg et al., 1997; Ivanov et al., 2016).

Inflammation, ROS production, and antioxidant depletion are related to each other: inflammatory cells release ROS at sites of inflammation, leading to an imbalance between oxidants and antioxidants in favor of the oxidants and subsequently to disruption of redox signaling, consequently resulting in oxidative stress, and without an efficient antioxidant response ROS or reactive nitrogen species (RNS) can activate intracellular signaling pathways that result in the expression of pro-inflammatory genes (Biswas, 2016). During ART, residual levels of viral replication are associated with persistent low-level inflammation, thus supporting HIV-1 reservoir replenishment and contributing to HIV-1 persistence (Deeks et al., 2013; Massanella et al., 2016). Besides, increased oxidative stress during HIV-1 infection, rising either directly from the virus or indirectly from HIV-related inflammation, supports the activation of latently HIV-1-infected cells (Ivanov et al., 2016). Less is known about the role of inflammation in EC, although some studies, including an earlier study from our group, suggest that gene expression and/or levels of inflammatory markers are low and comparable to HIV-uninfected individuals (Cortes et al., 2018; Hocini et al., 2019; Zhang et al., 2018). In contrast, one study reported increased levels of inflammatory markers in EC (Li et al., 2015). This observation could be due to the heterogeneous nature of the EC.

We observed that the metabolites that are associated with inflammation were reduced in EC, namely the kynurenine pathway of tryptophan metabolism (KP), where tryptophan is metabolized to kynurenine, in the liver by tryptophan dioxygenase (TDO) and in extra-hepatic tissues by the enzyme indoleamine 2,3-dioxygenase (IDO). The activity of the latter is induced by inflammatory cytokines, for example interferon-gamma (IFNγ) and tumor necrosis factor alpha (TNFα) (Figure 5A) (Hopper et al., 2012). Both kynurenine and its derivative and NAD precursor quinolinate displayed significant decreases in EC relative to VP, whereas VP showed trending (kynurenine) or significant (quinolinate) increases in these same molecules relative to HC (Figure 5B). At the same time, levels of serotonin and tryptophan were significantly lower in VP compared with EC and HC. These data suggest that VP exhibit increased metabolism of tryptophan to kynurenine, possibly due to inflammation. This observation matches with characteristically elevated inflammation markers in treatment-naïve HIV-1-infected individuals (Paiardini and Müller-Trutwin, 2013). Relative to VP, decreased activity of KP is observed in EC and that could be due to maintenance of physiological levels of inflammation and viral suppression in EC. We furthermore measured plasma levels of 83 soluble inflammation markers, and most of them were not statistically different between EC and HC, indicating a physiological state of inflammation in EC, too. That also applies to neopterin, which is not only a pro-inflammatory marker and associated with oxidative stress but has also been described as a diagnostic and prognostic marker in HIV-1 infection. Neopterin levels correlate positively with viral load, decrease ART, and predict HIV-1-related mortality (Eisenhut, 2013; Gieseg et al., 2018).

Interestingly, only levels of three cytokines (CCL4, CCL7, and CCL20) as well as NOS3 levels were higher in EC compared with HC. Not all of these proteins belong to classical inflammatory markers though, and for the three cytokines some antiviral activities have been reported. For CCL4 and CCL7 a similar trend was also seen in our previous study with more EC samples, although it was not statistically significant, revealing the heterogeneous characteristics of EC (Zhang et al., 2018). CCL4 and CCL7 both bind to HIV-1 co-receptor CCR5, with CCL4 being a known HIV-1-suppressive factor (for CCR5-tropic strains) by acting as a competitor to viral binding site (Blanpain et al., 1999). Our finding of increased CCL4 levels in EC is consistent with a study by Walker et al. (Walker et al., 2015). CCL20 was shown to have antiviral activity against HIV-1 in the female reproductive tract with a direct interaction of this chemokine with HIV-1 as the proposed mechanism of inhibition (Ghosh et al., 2009). Thus, elevated plasma levels in EC might contribute to virus control by acting as a direct antiviral (Lee and Körner, 2017).

In our earlier transcriptomics study, we observed decreased RNA levels of CCR5 in EC compared with HC in the same population (Zhang et al., 2018). To our surprise, we did not see significant changes in the number of cells with surface expression of CCR5. Instead, we observed a lower frequency of CCR6^+^ cells in all the subpopulations investigated in EC accompanied by reduced CCR6 surface levels on both CD4^+^ and CD8^+^ T cells. This finding is in accordance with a study by Gosselin et al. that reported diminished frequencies of CCR6^+^ T cells in HIV-1-infected subjects, both treatment-naïve and on ART. Thus, reducing CCR6^+^ T cells might be a consequence of HIV-1 infection, also happening in EC. The same study further revealed that, although reduced in frequency, the CCR6^+^ T cells harbored higher amounts of integrated HIV-1 DNA than CCR6^−^ T cells. CCR4^+^CCR6^+^ and CXCR3^+^CCR6^+^ T cells were furthermore highly permissive to HIV-1 replication, regardless of virus tropism (CCR5 or CXCR4), tested both in a cell line and in primary T cells (Gosselin et al., 2010). The CCL20/CCR6 axis has earlier been discussed in the context of HIV-1 infection. Damaged epithelial surfaces at sites of HIV-1 infection lead to release of CCL20 having anti-HIV-1 properties. CCL20 attracts CCR6^+^ cells such as dendritic cells (DCs) and CD4^+^ T_H_17 cells that migrate along the CCL20 gradient to the site of infection. These cells might get infected and disseminate infection further to lymph nodes, where even more CD4^+^ cells can be infected. Because CCR6^+^ were seen to be highly permissive for HIV-1 replication and harbor HIV-1 DNA, they might particularly support HIV-1 persistence and dissemination. Thus, CCL20/CCR6 is considered to be a “double-edged sword” concerning HIV-1 infection (Lee and Körner, 2017). In our study, CCL20 plasma levels were increased, whereas CCR6 expression was decreased on circulating CD4^+^ and CD8^+^ T cells in EC (frequency and surface expression levels). Therefore, it seems like the CCL20-CCR6 interplay per se is not increased in EC. Albeit, it cannot be excluded that CCR6^+^-expressing cell frequencies in EC peripheral blood are low because they infiltrate in other tissues along CCL20 gradient, where they support virus replication, as it might be the case in untreated viral progressors (Gosselin et al., 2010). That needs further clarification.

The chemokine CCL7 can bind to CCR1 (not included in our analyses), CCR2, and CCR3, as well as (as an antagonist) to CCR5. No changes were seen in the expression of the CCR3 and CCR5 receptors. However, the frequency of CCR2-expressing CD8^+^ T cells was reduced in EC compared with HC. As of today, HIV-1-related research has mainly focused on CCR2 expression on monocytes, including a study that reported reduced proportions of CCR2^+^ monocytes in EC. Further, a higher proportion of intermediate monocytes was observed in EC in the same study (Krishnan et al., 2013). We did not see any of these two features in our cohort. Recently, reduced frequencies and surface levels of CCR2 on CD4^+^ T cells were described in a subset of EC samples (but not in all) (Gonzalo-Gil et al., 2019). Although we did not see any difference in frequencies of CCR2^+^ CD4^+^ T cells, we noticed a reduction of CCR2 surface expression levels on CD4^+^ T cells in EC. Furthermore, frequencies and surface levels of CCR2 on CD8^+^ T cells were diminished in EC. So far, the role of CCR2 expression on CD8^+^ T cells, especially in HIV-1 infection, remains mostly elusive.

NOS3 was the fourth plasma protein with higher levels in EC relative to HC, but it was the only one not being a chemokine. NOS3 and other nitric oxide synthases convert the amino acid L-arginine and oxygen into L-citrulline and nitric oxide (NO) in a complex oxidoreductase reaction. NOS3 can also contribute to limiting immune responses regulating inflammatory processes. *In vitro* studies reported stimulatory and inhibitory effects of hydrogen sulfide (H_2_S) on expression and activity of NOS3, with H_2_S deriving from homocysteine or cysteine, thus creating a link to the transsulfuration pathway and glutathione metabolism, as discussed earlier. It is, however, unclear whether and how H_2_S affects NOS3 *in vivo* (Bogdan, 2015). The biological impact of elevated NOS3 levels in EC is unclear and needs further studies. Due to its immunomodulatory functions, it could be speculated that increased levels of NOS3 contribute to reduced inflammation in EC.

Even though CCL4, CCL7, and CCL20, as well as NOS3 had higher levels in EC compared with HC in our study, it seems like these proteins rather contribute to EC control state instead of having pathophysiological inflammatory effects. In contrast, NOS3 even appears to have an inflammation-reducing function; however, its exact role is unclear. Altogether, low-level inflammation and physiological antioxidant defense levels might contribute to control viral replication and viral reservoir in EC. A central question remaining is whether EC, due to functioning antioxidant defense mechanisms, exhibits lower oxidative stress compared with viremic progressors and has, therefore, less inflammation, thus creating an unfavorable environment for viral replication or vice versa whether low-level inflammation is supportive for antioxidant defense stemming HIV-1 pathophysiology. Both approaches shall be considered in HIV-1 cure strategies.

One possible link between inflammation, oxidative stress, and antioxidant defense could be the oxidative phosphorylation system. It is located in the mitochondria and consists of the electron transport chain as well as ATP synthase and is responsible for mitochondrial respiration and ATP production. Hence, it is a crucial part of the cells' energy metabolism. Oxidative phosphorylation involves oxygen and the production of ROS that, under physiological conditions, is counteracted by antioxidant defense systems. Rising numbers of immunometabolic studies in the field of HIV-1 describe increased mitochondrial respiration in CD4^+^ T cells of HIV-1-infected persons with glutamine as a source for oxidative phosphorylation. These changes in immunometabolism are associated with increased expression of ROS and inflammatory cytokines, and immunometabolic dysfunction might further mediate the development of age-related diseases (Butterfield et al., 2020; Spinelli and Haigis, 2018). Further investigating the pathway of oxidative phosphorylation might give valuable insights into HIV-1 pathogenesis, and targeting it with drugs could have a promising therapeutic potential for HIV-1-infected individuals.

To summarize, low-level inflammation compared with VP and physiological antioxidant defense levels observed in our EC cohort might be an essential factor contributing to EC phenotype. Earlier studies showed that HIV-infected cells had altered antioxidant defenses, and the impact could be variable depending upon the stages of the viral life cycle (Sandstrom et al., 1998). Exploring and unraveling the processes of inflammation, oxidative pathways, and antioxidant defense, as well as their implication in HIV-1 infection, is of great importance for developing new therapeutic strategies.

### Limitations of the study

Our study has a few limitations that merit comments. Due to their rare occurrence, the number of EC is relatively low. However, this is one of the largest EC cohorts, which has more than median of 10 years of HIV-1 positivity without any treatment. Due to the recent treatment guideline for “treat-all,” it is difficult to identify therapy-naïve EC. In order to have study groups as homogeneous as possible (age-, BMI-, and gender-matched) number of EC samples and consequently also of the other two groups were even more reduced. Also, proteomics analyses were of targeted nature and did not provide a complete picture of the plasma proteome.

### Resource availability

#### Lead contact

Further information and requests for resources and reagents should be directed to and will be fulfilled by the Lead Contact, Ujjwal Neogi (ujjwal.neogi@ki.se).

#### Materials availability

This study did not generate unique reagents.

#### Data and code availability

All metabolomics data associated with this study are present in this paper including the Supplementary Materials. Proteomics data presented in this paper or Zhang et al. (2018). The flow cytometry data are available at the flow repository (repository ID FR-FCM-Z2T3). The codes used during this study are available at GitHub (https://github.com/neogilab/METABO-EC). The original scale metabolomics data are available from https://doi.org/10.6084/m9.figshare.13585955.

## Methods

All methods can be found in the accompanying Transparent Methods supplemental file.

## Supplemental information

Document S1. Transparent methods, Figures S1–S9, and Table S1
