# Supplementary material for: Distinct lipid profile, low-level inflammation, and increased antioxidant defense signature in HIV-1 elite control status
Source: iScience. 2021 Jan 28;24(2):102111. doi: 10.1016/j.isci.2021.102111 (PMC7892918; doi:10.1016/j.isci.2021.102111)
Supplement: Document S1. Transparent methods, Figures S1–S9, and Table S1 [file mmc1.pdf]

iScience, Volume *24*

Supplemental Information

Distinct lipid profile, low-level inflammation, and increased antioxidant defense signature in HIV-1 elite control status

Maike Sperk, Flora Mikaeloff, Sara Svensson-Akusjärvi, Shuba Krishnan, Sivasankaran Munusamy Ponnan, Anoop T. Ambikan, Piotr Nowak, Anders Sönnerborg, and Ujjwal Neogi

Supplemental Information

Fig. S1. Unsupervised PCA of all samples with all metabolites related to Fig 1. All the samples cluster together except EC06 that was well-separated and is there classified as an outlier. HIV-1 elite controllers are marked orange, HIV-negative control green, and viremic progressors red.

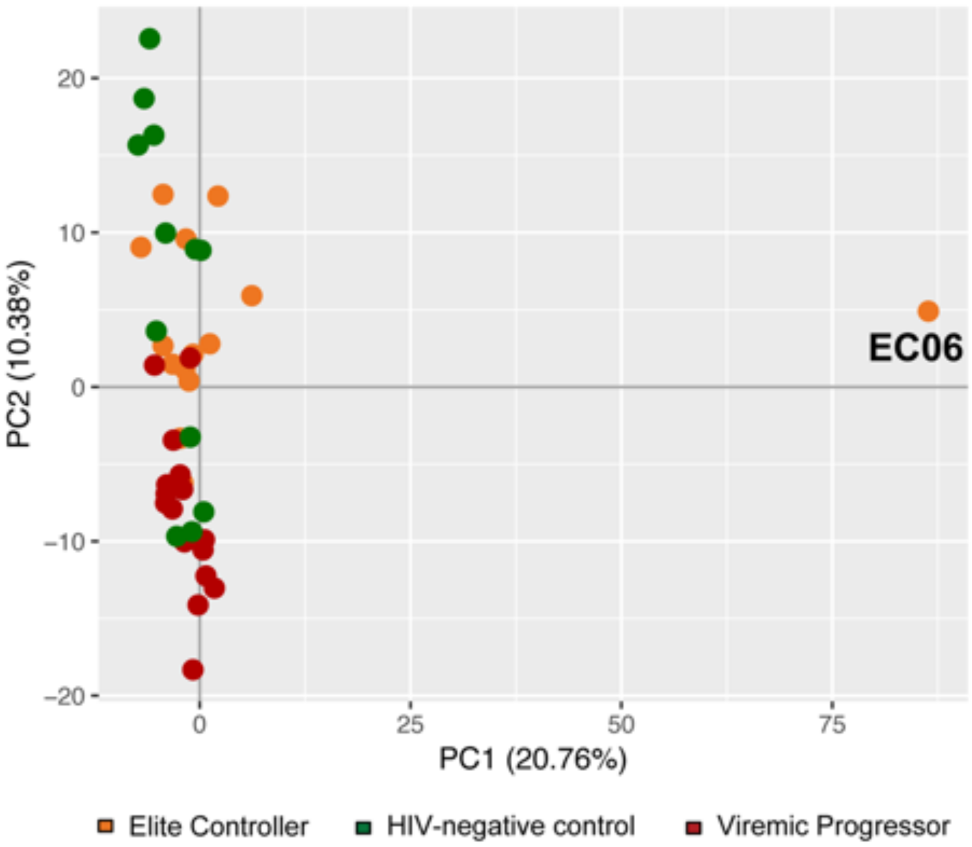

Fig. S2. Differential expression of the metabolomics data related to Fig 1. (**A-C**) Differential expression of the metabolomics data is presented as volcano plots for (A) EC vs VP, (B) VP vs HC, and (C) EC vs HC. Filled red circles are used for metabolites with log2-foldchange smaller than -1.0 and adjusted p-value<0.05 (equals adj. p-value greater than $-\log_{10}1.3$). Filled green circles picture metabolites with log2-foldchange greater than 1.0 and adjusted p-value<0.05. Black filled circles constitute metabolites with log2-foldchange smaller than 1.0 and adjusted p-value<0.05. Grey filled circles are metabolites with adjusted p-value>0.05.

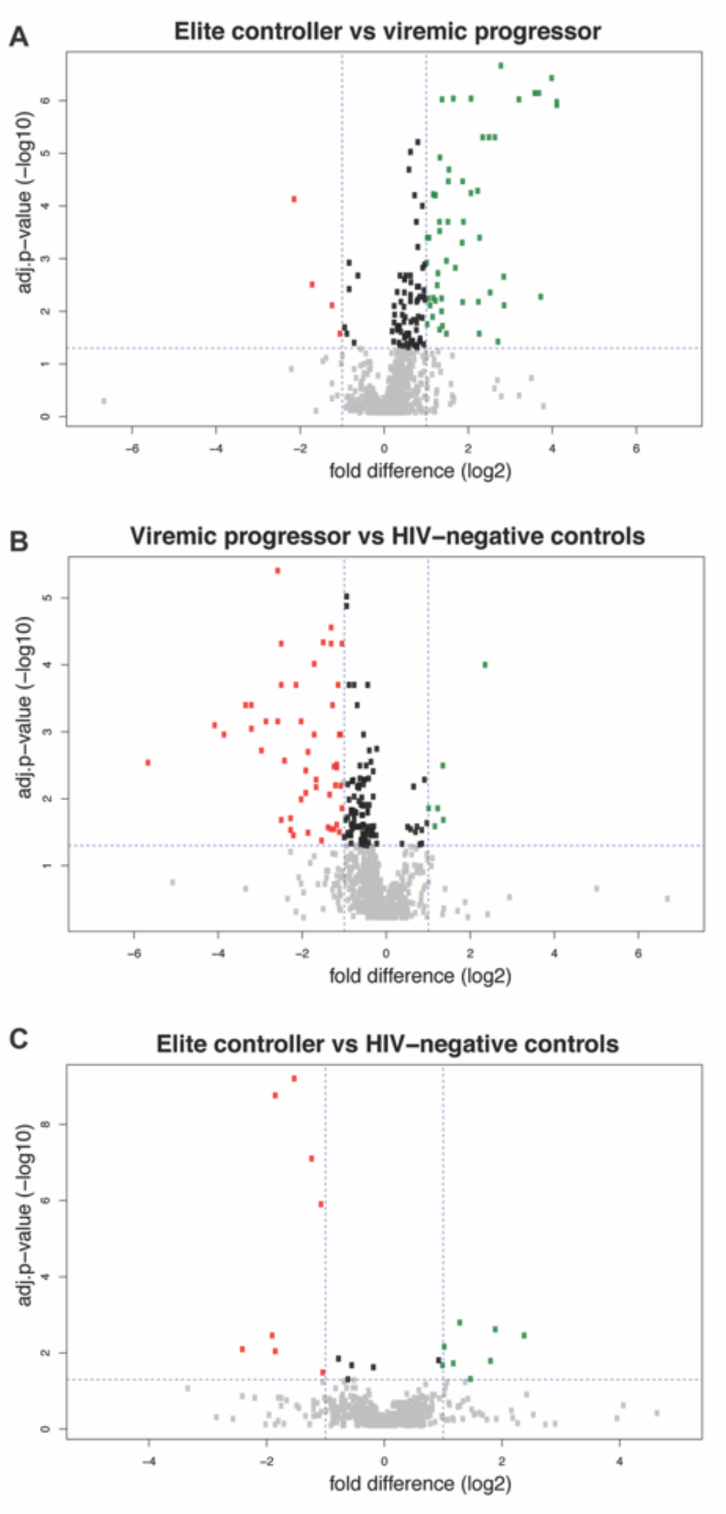

Fig. S3. Random Forest (RF) analyses related to Fig 2.
Random Forest (RF) analyses showing the top 30 metabolites that contribute to separation between (**A**) EC and HC, (**B**) HC and VP, as well as (**C**) EC and VP. Affiliation of each metabolite to a certain super pathway is marked by different colours (see colour-coded legend). Biochemicals involved in lipid and amino acids metabolism dominate the top-ranked intermediates for all three comparisons. The table represents predictive accuracy, predicted and actual grouping of the samples.

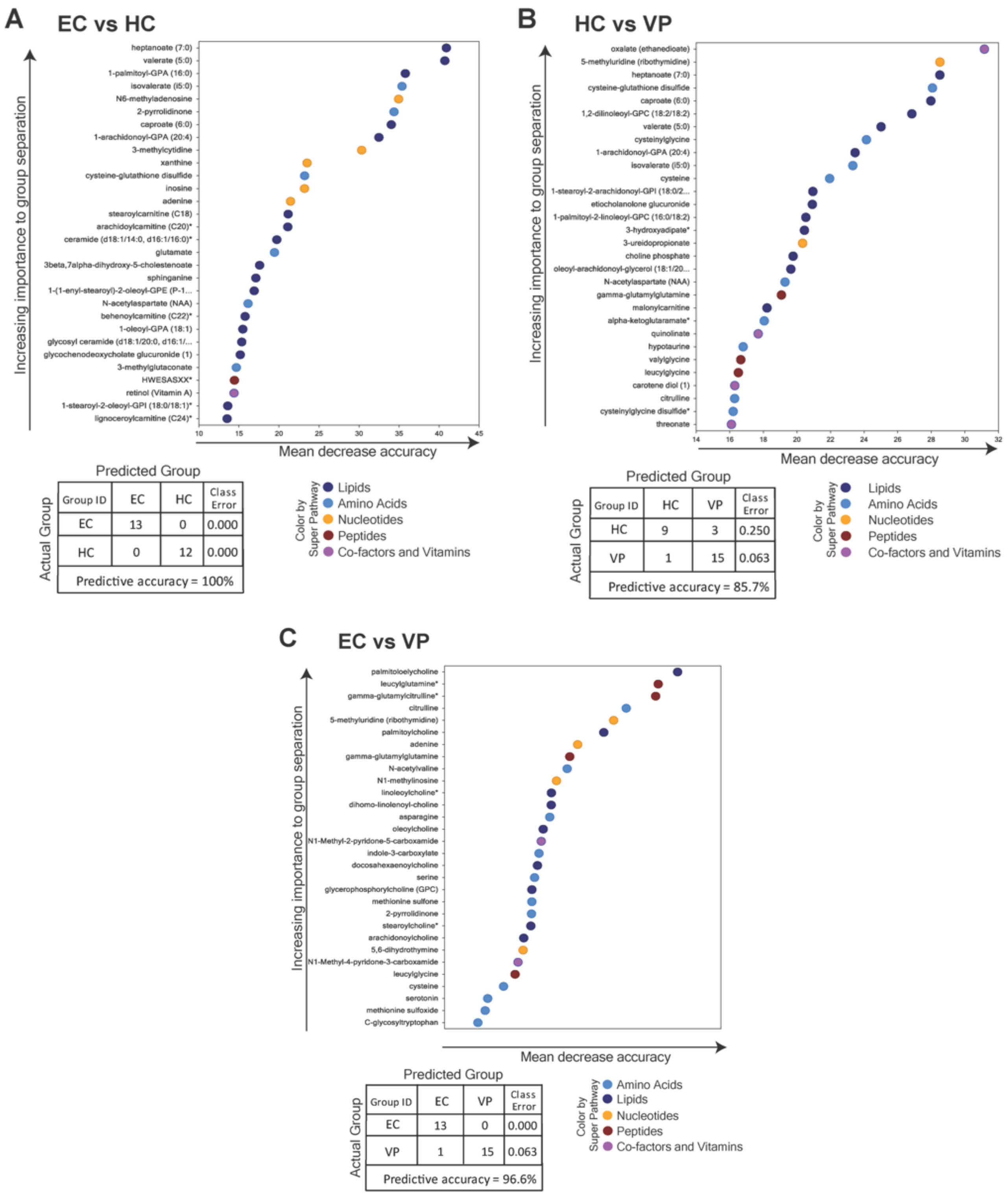

Fig. S4. Network analyses of the significantly different metabolites related to Fig 3.
(**A** and **B**) Network analyses of the metabolites that were significantly different in (A) EC vs VP (236 metabolites) and (B) VP vs HC respectively (256 metabolites). Rectangular nod shapes represent the eight super pathways that are shown in different colours according to legend. Octagonal nod shapes are used for sub pathways belonging to the eight super pathways. Circular nod shapes show the single metabolites, where red indicates increased levels and green indicates decreased levels. Size of the circles picture p-value: the bigger the size the lower the p-value. Lines connect each metabolite to its respective sub pathway and sub pathway to their respective super pathways.

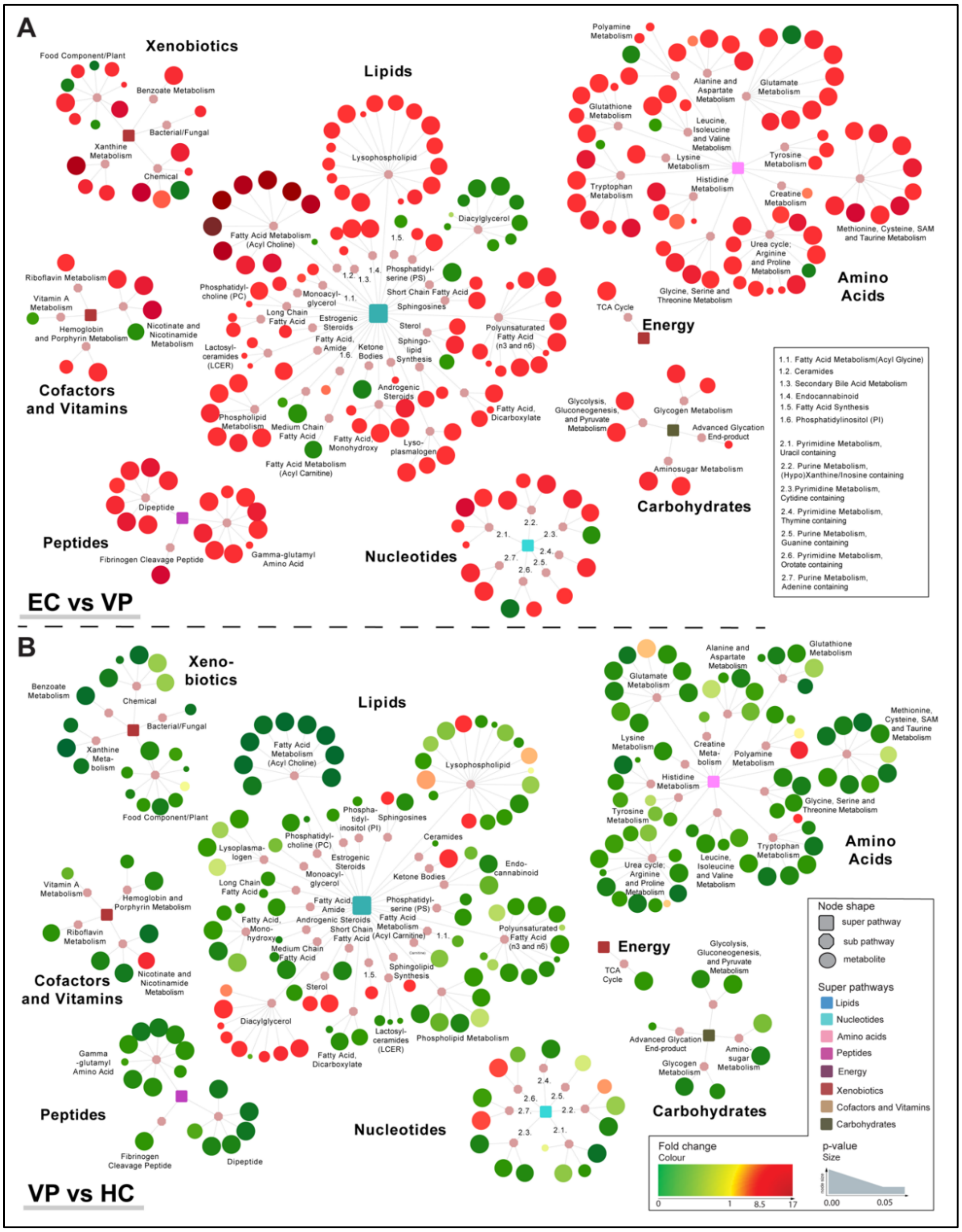

Fig. S5. Methionine, transsulfuration, and glutathione metabolism related to Fig 4.
Boxplots showing scaled intensity of 16 selected metabolites that play a role in methionine, transsulfuration, or glutathione metabolism. For all selected biochemicals, EC (orange) have higher levels relative to VP (red); and HC (green) has higher levels than VP for almost all metabolites shown, based on ANOVA contrasts with *indicating p-value<0.05, **p-value<0.01, and ***p-value<0.001 respectively. Median values and interquartile ranges are indicated by bars.

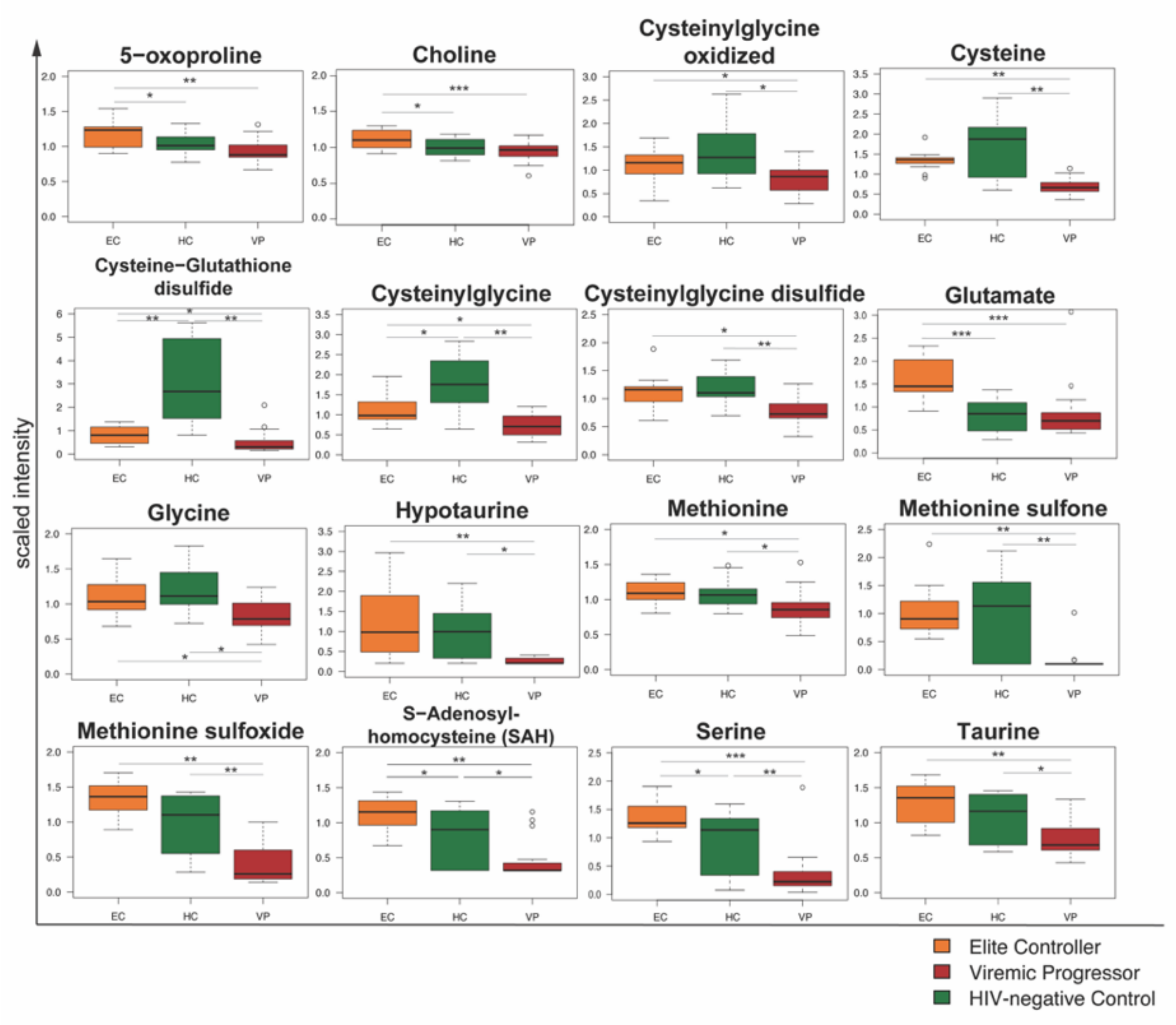

Fig. S6. Detection of ROS levels and gene expression of NRF2-ARE genes related to Fig 4.
(**A**) Dot plots representing MFI of total ROS in PBMC sub-populations ($CD4^+$ and $CD8^+$ T-cells, classical, intermediate and non-classical monocytes measured by FACS of EC (orange) and HC (green) samples. Mann-Whitney test was used to calculate p-values, no significant difference was observed. Median values are indicated by bars.
(**B**) Dot plots representing gene expression of NRF2-ARE genes in EC (orange) and HC (green). Mann-Whitney test was used to calculate p-values, no significant difference was observed. Median values are indicated by bars.

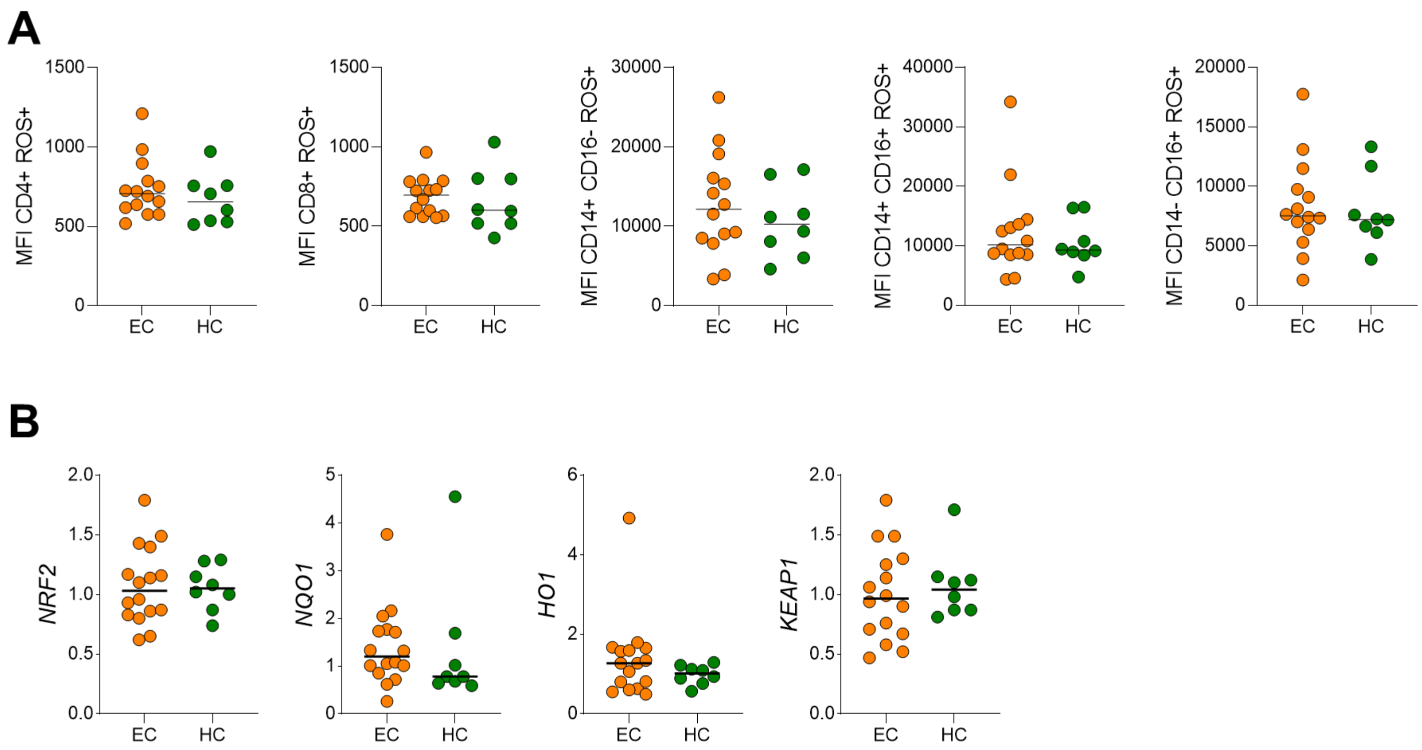

Fig. S7. Boxplots representing plasma levels of CRP and Neopterin determined by ELISA related to Fig 5. (**A** and **B**) Boxplots representing plasma levels of (A) CRP and (B) Neopterin determined by ELISA for EC (orange), HC (green), and VP (red). Concentrations are given according to unit on y-axis. Mann-Whitney test was used to calculate p-values with *p-value<0.05, **p-value<0.01, ***p-value<0.001. Median values and interquartile ranges are indicated by bars.

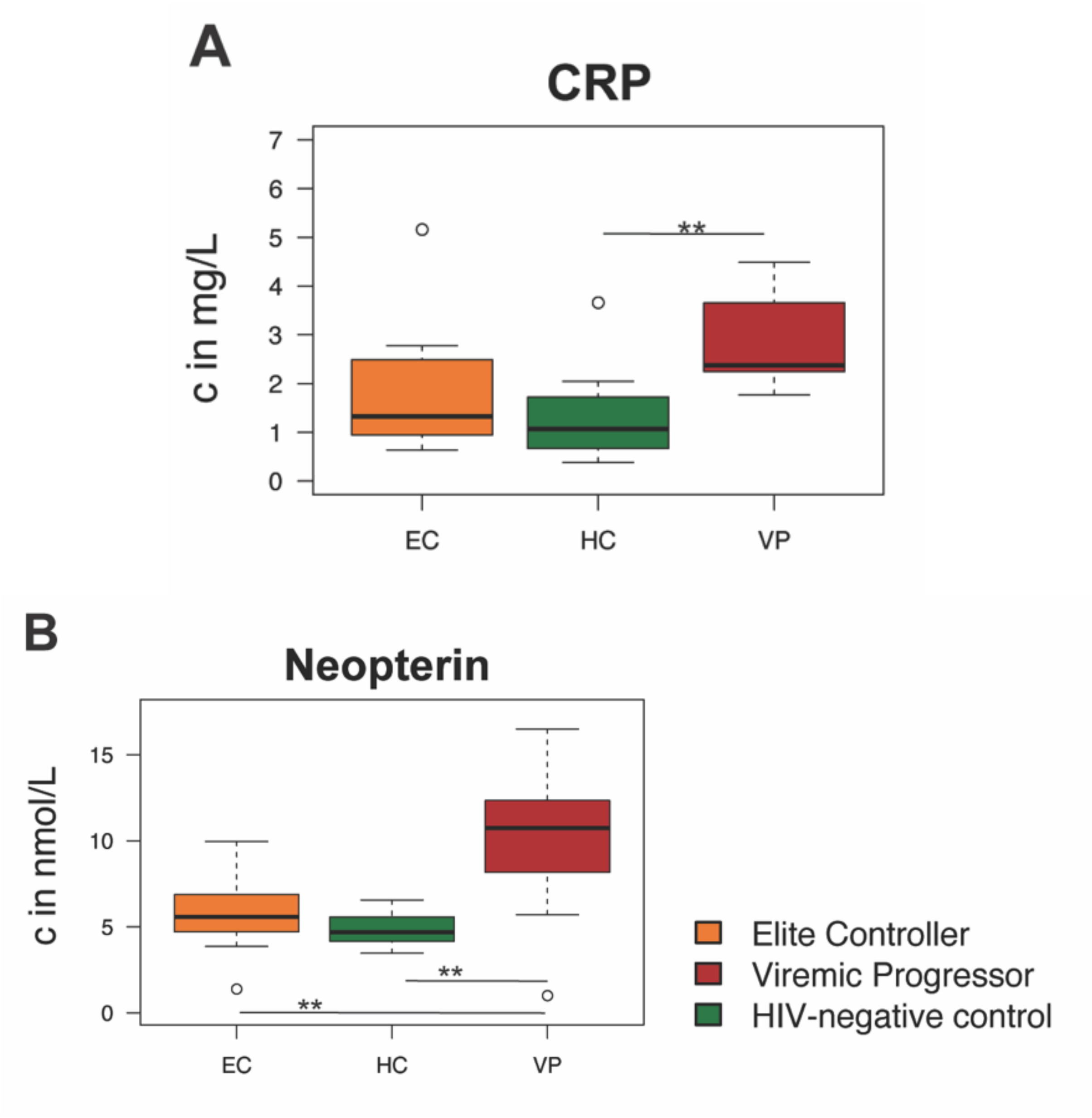

Fig. S8. Additional figures of flow cytometry data analyses related to Fig 6.
(**A**) Boxplots represent expression frequency of selected surface receptors on different cell populations for EC (orange) and HC (green). Median values and interquartile ranges are indicated by bars. P-values are determined by Mann-Whitney U test with * indicating p-value<0.05 and **p-value<0.01. (**B**) t-SNE plots illustrating differences between EC (upper plot) and HC (lower plot) in surface expression of the nine targeted surface markers CD3, CD4, CD8, CD14, CD16, CCR2, CCR3, CCR5, and CCR6 respectively. The plots show t-SNE dimension 1 and t-SNE dimension 2. Blue colour shows low expression, green intermediate expression, and red high expression. Prior analysis, viable singlet cells of each sample were downsampled to 10,000 and individual downsampled samples of EC and HC respectively were concatenated. t-SNE analysis was performed with 2,000 iterations with perplexity of 20 and learning rate of 1,000.

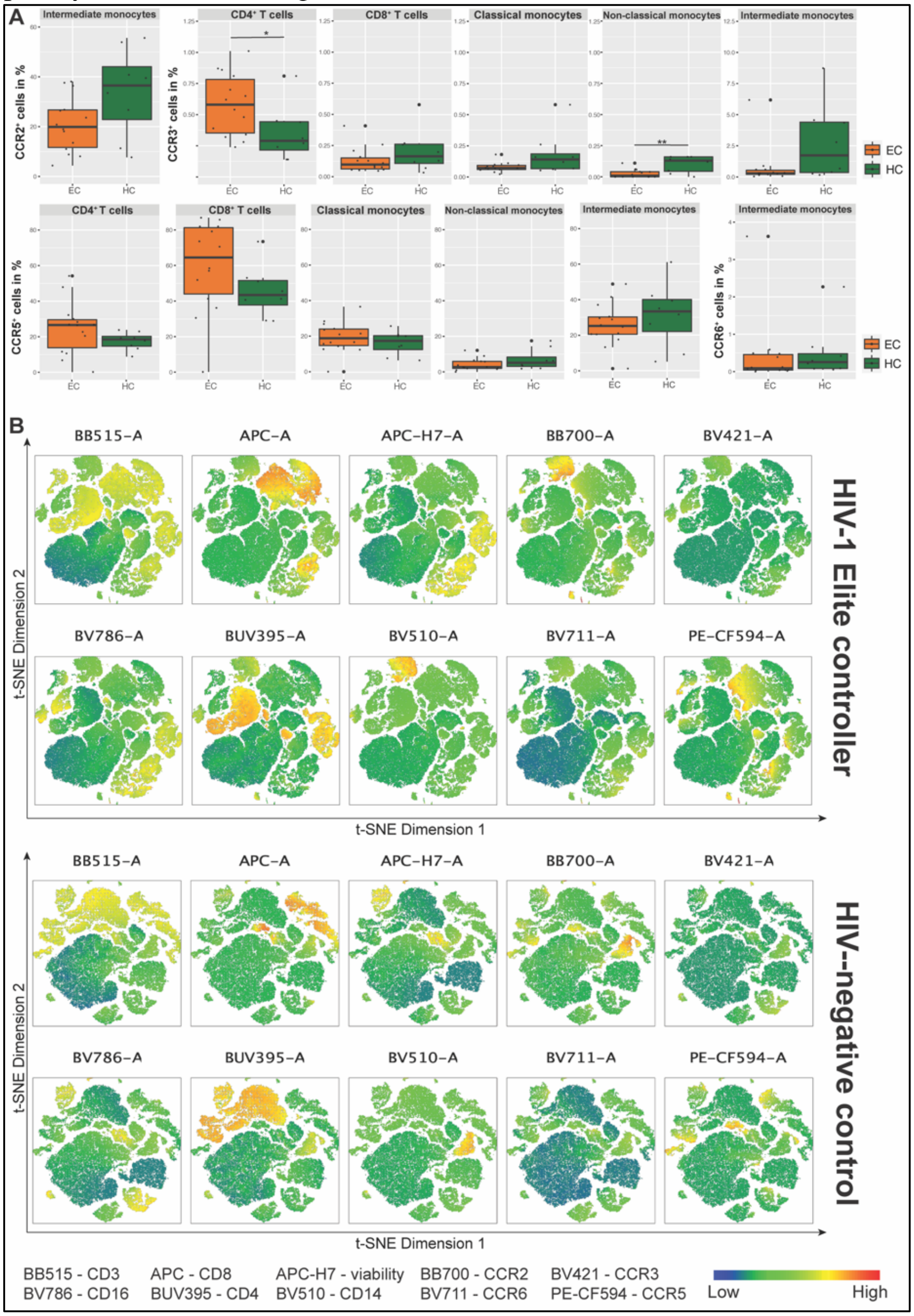

Fig. S9. Gating strategy of flow cytometry data related to Fig 6.

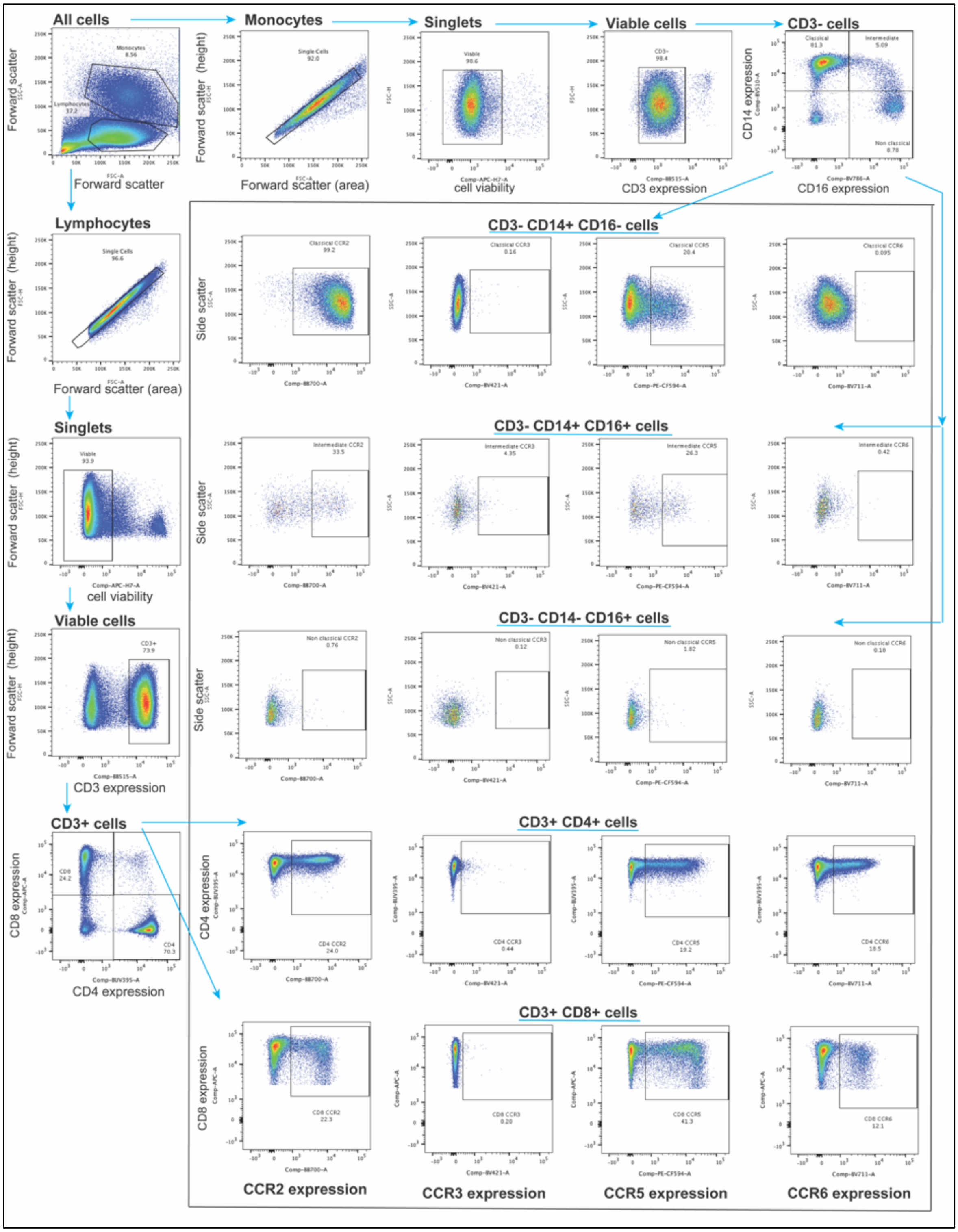

Table S1. Gamma-glutamyl amino acids related to Fig 4. Table with 17 metabolites belonging to the sub pathway of gamma-glutamyl amino acids that have been detected in metabolomics analyses. Foldchange between two study groups is given in column 3-5 with (EC vs HC, VP vs HC, and EC vs VP) with respective p-value and q-value in rows 6-11. Foldchange written in yellow numbers indicates statistical significance (p-value<0.05) with green cells marking downregulation and red cells marking upregulation. ANOVA contrasts were used to calculate p-values as well as q-values.

		EC / HC	VP / HC	EC / VP	EC / HC		VP / HC		EC / VP	
					p-value	*q*-value	*p*-value	*q*-value	*p*-value	*q*-value
Gamma-glutamyl Amino Acid	gamma-glutamylalanine	0,74	0,93	0,80	0,8396	0,7597	0,2943	0,3069	0,1994	0,4138
	gamma-glutamylglutamate	1,44	0,72	1,99	0,1498	0,4043	0,0405	0,0982	0,0006	0,0060
	gamma-glutamylglutamine	1,14	0,36	3,18	0,3049	0,5370	0,0000	0,0000	0,0000	0,0000
	gamma-glutamylglycine	1,09	0,60	1,83	0,5986	0,6679	0,0029	0,0181	0,0005	0,0053
	gamma-glutamylhistidine	0,99	0,81	1,21	0,8598	0,7687	0,0675	0,1305	0,0410	0,1473
	gamma-glutamylisoleucine	1,08	0,96	1,12	0,3872	0,5681	0,9177	0,5780	0,4109	0,6104
	gamma-glutamylleucine	0,93	0,90	1,04	0,7045	0,7203	0,4278	0,3788	0,6841	0,7543
	gamma-glutamyl-alpha-lysine	0,96	0,74	1,30	0,8940	0,7813	0,0181	0,0566	0,0223	0,0948
	gamma-glutamyl-epsilon-lysine	1,14	0,96	1,20	0,3125	0,5391	0,6012	0,4591	0,1100	0,2925
	gamma-glutamylmethionine	1,03	0,65	1,57	0,6890	0,7111	0,0007	0,0063	0,0001	0,0021
	gamma-glutamylphenylalanine	0,93	0,97	0,96	0,5009	0,6269	0,6362	0,4763	0,8109	0,7932
	gamma-glutamylthreonine	1,14	0,57	1,98	0,3026	0,5343	0,0025	0,0164	0,0001	0,0013
	gamma-glutamyltryptophan	1,03	0,71	1,45	0,7795	0,7421	0,0005	0,0052	0,0002	0,0021
	gamma-glutamyltyrosine	1,04	0,99	1,05	0,8064	0,7513	0,7870	0,5288	0,5904	0,7162
	gamma-glutamylvaline	1,04	1,08	0,97	0,7799	0,7421	0,3307	0,3256	0,4846	0,6664
	gamma-glutamylcitrulline	1,32	0,31	4,26	0,1526	0,4060	0,0000	0,0001	0,0000	0,0000
	gamma-glutamyl-2-aminobutyrate	1,05	0,83	1,27	0,6107	0,6698	0,4100	0,3688	0,1689	0,3861

TRANSPARENT METHODS

Human subjects

Human plasma samples were obtained from three groups of individuals: untreated HIV-1-infected individuals with viremia (viremic progressor, VP, n=16) or without viremia (EC, n=14) and HIV-negative individuals (herein mentioned as HIV-negative controls, HC, n=12). Study groups consist of female and male individuals and have comparable gender proportions. Samples are age- and BMI-matched and are part of the InfCareHIV cohort from Karolinska University Hospital, Huddinge, Sweden. Peripheral blood mononuclear cells (PBMCs) were used from a subpopulation of these subjects (EC n=14 and HC n=8). EC was defined as being infected with HIV-1 for more than a year with ≥3 consecutive viral loads (VL) <75 RNA copies/ml blood (and all previous VL<1000 RNA copies/ml) or as having a known HIV-1-infection for at least ten years with two or more VL measurements of which 90% were below 400 RNA copies/mL. Patients' characteristics are given in Table S2.

The study is approved by the Swedish Ethical Review Authority with ethical permit Dnr 2013/1944-31/4 and amendment Dnr 2019-05585. Written informed consent was obtained from all study participants prior to inclusion in the study. Patients identities were anonymized before analysis.

Table S2. Patients' clinical and demographic characteristics related to Fig 1A. P-values were determined by either (a) Kruskal Wallis Test or (b) chi square test. Routes of sexual transmissions are: transmission through blood donation product (BDPT), heterosexual transmission (HET), men who have sex with men (MSM), other transmission route (OTR), people who inject drugs (PWID), information not available (NA). BMI, body mass index.

	EC	VP	HC	p-value
**Sample size, n**	14	16	12	
**Age, median (IQR)**	45.5 (40.25-50.50)	45.0 (40.75-53.25)	44.00 (42.00-46.50)	0.7008 (a)
**Gender, female, n**	7 (50)	7 (43.75)	6 (50)	
**BMI, median (IQR)**	26.40 (24.80-32.23)	26.00 (23.75-30.50)	23.00 (21.45-25.50)	0.0537 (a)
**Route of transmission / Sexual orientation, n (%)**				
**BDPT**	2 (14.29)			0.393 (b)
**HET**	8 (57.14)	10 (62.5)	5 (41.67)	
**MSM**	3 (21.43)	4 (25.00)	-	
**OTR**	1 (7.14)	-	-	
**PWID**	-	2 (12.5)		
**NA**	-	-	7 (58.33)	
**HIV-1 subtype, n (%)**				
**A**		2 (12.5)		0.5509 (b)
**A1**		1 (6.25)		
**B**	2 (14.29)	2 (12.5)		
**C**	4 (28.57)	3 (18.75)		
**CRF**	1 (7.14)	2 (12.5)		
**NA**	7 (50)	6 (37.5)		

METHOD DETAILS

Metabolomics

Metabolomic profiling was performed at Metabolon, Inc. (North Carolina, USA) with non-targeted mass spectrometry (MS) analysis as described recently (Babu et al., 2019). Briefly, samples were first prepared with an automated MicroLab STAR system (Hamilton Company) and then analyzed by four ultra-high-performance liquid chromatography-tandem mass spectrometry according to Metabolon pipeline. Biochemical components were identified based on retention time/index (RI), mass to charge ratio (m/z), and chromatographic data (MS/MS spectral data) by comparison to the Metabolon reference library that consists of more than 3300 compounds. The method is ISO 9001:2015 certified and the lab is accredited by the College of American Pathologist (CAP), USA. The metabolites were mapped with the life-style and environmental factors as reported recently (Bar et al., 2020).

Network analyses

Metabolites and pathways networks were created with Cytoscape ver 3.6.1(Shannon et al., 2003). For each metabolite, fold change and p-value and q-value from the Mann-Whitney U test were added to network template file. Nodes are connected objects in the network and edges connections between nodes. Metabolites are linked to subpathways and subpathways to superpathways. Gradient color and size were applied to metabolites nodes depending on fold-change.

Targeted plasma proteomics

Plasma samples were analyzed using proximity extension assay (PEA) as described earlier(Zhang et al., 2018). Olink Immuno-Oncology panel was applied that includes 92 plasma proteins (Olink Bioscience, RRID:SCR_003899, Cat# 95310). In addition, enzyme-linked immunosorbent assay (ELISA) was performed for neopterin (TECAN, Cat# Cat# RE59321), C-reactive protein (CRP, R&D systems, Cat# DCRP00), and CCL20 (R&D Systems, Cat# DM3A00) as per manufacturer’s instruction.

Flow cytometry

PBMCs of 14 EC and 8 HC were subjected to flow cytometry analyses. Samples were thawed, washed with flow cytometry buffer (PBS with 2% fetal bovine serum and 2mM EDTA) and stained for cell surface markers for 20 minutes at room temperature. The following antibodies were used: FITC mouse anti-human CD3 (BioLegend Cat# 317306, RRID:AB_571907), APC mouse anti-human CD8a (BioLegend Cat# 301014, RRID:AB_314132), PerCP anti-human CD8a (BioLegend Cat# 300922, RRID: AB_1575072), Brilliant Violet 510 mouse anti-human CD14 (BioLegend Cat# 301842, RRID:AB_2561946), Brilliant Violet 421 mouse anti-human CD193 (CCR3) (BioLegend Cat# 310714, RRID: AB_2561886), and Brilliant Violet 711 mouse anti-human CD196 (CCR6) (BioLegend Cat# 353436, RRID:AB_2629608) as well as BUV395 mouse anti-human CD4 (BD Biosciences Cat# 563550, RRID:AB_2738273), BV786 mouse anti-human CD16 (BD Biosciences Cat# 563690, RRID:AB_2744299), BB700 mouse anti-human CCR2 (BD Biosciences, Cat# 747847, RRID:AB_2861365),

and PE-CF594 Mouse Anti Human CD195 (CCR5) (BD Biosciences Cat# 562456, RRID: AB_11154599). All stainings were complemented with Live/Dead fixable near IR dye (Invitrogen, Cat# L10119). Cellular ROS levels were measured using CellROX™ Deep Red Flow Cytometry Assay Kit according to manufacturer's instructions (Invitrogen, Cat# C10491). A detailed list of antibodies used is provided in the key resources table. After antibody incubation, cells were washed with flow cytometry buffer and fixed with 2% paraformaldehyde for 15 minutes at room temperature. Acquisition was performed on BD FACS Symphony (BD Bioscience, USA) using lasers and filter settings as indicated for BUV395, BV421, BV510, BV711, BV786, PerCP, FITC, BB700, PE-CF594, APC and Near IR respectively; 355nm UV (100mW) 379/28, 405nm violet (100mW), 450/50, 525/50 (505 LP), 710/50 (685 LP), 810/40 (770 LP), 488nm blue (200mW) 670/30 (635 LP), 530/30 (505 LP), 710/50 (685 LP), 561nm Y/G (200mW), 610/20 (600LP) and 637nm Red (140mW), 670/30 and 780/60 (750LP). Flow cytometry analysis was performed using FlowJo 10.6.2 (TreeStar, Inc, Ashland, OR) and visualization of complex data using Spice(Roederer et al., 2011). A gating strategy is provided in Fig. S9. Expression frequency and levels of selected surface markers are represented as boxplots using ggplot2 R packages. T-distributed Stochastic Neighbor Embedding (t-SNE) analysis was conducted in FlowJo 10.6.1 with 2,000 iterations, 20 perplexities and a learning rate of 1,000.

RT-qPCR Analysis

RNA was extracted using TRI Reagent (Zymo Research, Cat# R2050-1-200) and Direct-zol™ RNA Miniprep kit according to manufacturer's instructions (Zymo Research, Cat# R2050, R2052). cDNA was synthesized using SuperScriptTM IV reverse transcriptase (Thermofisher Scientific, Cat# 18090010), supplemented with Random Hexamer Primer (Thermofisher Scientific, Cat# SO142), dNTP Mix (Thermofisher Scientific, Cat# R0192) and RNasin® Ribonuclease Inhibitor (Promega, Cat# N2511). qPCR reactions were performed using KAPA SYBR Fast qPCR kit (KAPA Biosystems, Cat# KK4602) with primers targeting *NRF2 (*Fwd: 5'-CACATCCAGTCAGAAACCAGTGG-3' and Rev: 5'-GGAATGTCTGCGCCAAAAGCTG-3')*, KEAP1 (*Fwd: 5'-GAGCGCCTGGACGTAGAACCG-3' and Rev: 5'-GCTGCGAGTCCGAGGTCTTCC-3')*, HO1 (*Fwd: 5'-ACAAGGAGAGCCCAGTCTTC-3' and Rev: 5'-AGACAGGTCACCCAGGTAGC-3')*, NQO1 (*Fwd: 5'-CCTCTATGCCATGAACTT-3' and Rev: 5'-TATAAGCCAGAACAGACTC-3') and Actin (Fwd: 5'-GAGGGAAATCGTGCGTGACA-3' and Rev: 5'-AATAGTGATGACCTGGCCGT-3') on an ABI Fast 7500 system (Applied Biosystems) according to manufacturer's protocol. Gene expression analysis was performed using the ΔΔCT method and visualized using GraphPad Prism 8 (GraphPad Software Inc.).

Data visualization

Figures were prepared with Adobe Illustrator v24.

Statistical analyses of metabolomics data

Doughnut charts with detected metabolites were created in Microsoft Excel. One-way ANOVA was performed to identify biochemicals that differed notably between groups ($p \leq 0.05$). For multi-group comparison, a false discovery rate (FDR) adjusted $q \leq 0.05$ was utilized. In unsupervised analyses, volcano plots were used to visualize similarities and differences between sample groups. Further, principal component analyses (PCA) were performed to picture how individual samples differ from each other. The supervised classification technique Random Forest (RF) was applied to determine which biochemicals make the largest contribution to group classification. Based on the top 30 ranked metabolites from RF analyses that contribute to group separation, supervised hierarchical clustering analyses (HCA) were performed in R with gplots R package(RCoreTeam, 2020; Warnes et al., 2005) and a heatmap was generated by Pearson distance method. Supervised PCA was executed in R by ggplot2 R package(RCoreTeam, 2020; Wickham, 2016). Correlation analyses of metabolomics data were performed in R (RCoreTeam, 2020) with stats package, spearman correlations between specific sets of metabolites in EC. Boxplots of selected metabolites that are included in the correlation analyses were created in R(RCoreTeam, 2020) and show median and interquartile range.

Data exclusion

Regarding the metabolomics data, the sample EC06 was classified as an outlier and was excluded from further analyses of the metabolomics data. See also Figure S1.

Statistical analyses of proteomics data

Given data distribution, non-parametric Kruskal–Wallis H test was applied to extract proteins differing at least two groups (false discovery rate (FDR) < 0.05). Partial Least Squares Discriminant Analysis (PLS-DA) was performed using selected proteins using R package ropls(RCoreTeam, 2020; Thévenot et al., 2015). Two-sided Mann Whitney U Tests were carried out using R between two different conditions (FDR < 0.05). Identified proteins are represented as boxplots using ggplot2 R package(RCoreTeam, 2020; Wickham, 2016) showing median and interquartile range.

Statistical analyses of flow cytometry data

Statistical analyses of flow cytometry data were performed with Prism 8 (GraphPad Software Inc.). Two-sided Mann-Whitney U Test was used to make statistical comparisons between two study groups. Boxplots were created in R using ggplot2 R package(RCoreTeam, 2020; Wickham, 2016) and show median and interquartile range.

Statistical details

Statistical details of the experiments can be found in the figure legends. Significance was defined as p-value<0.05.

KEY RESOURCES TABLE

REAGENT or RESOURCE	SOURCE	IDENTIFIER
**Antibodies**		
FITC mouse anti-human CD3 (clone OKT3)	BioLegend	Cat# 317306, RRID:AB_571907
BUV395 mouse anti-human CD4 (Clone SK3)	BD Biosciences	Cat# 563550, RRID:AB_2738273
APC mouse anti-human CD8a (clone RPA-T8)	BioLegend	Cat# 301014, RRID: AB_314132
PerCP anti-human CD8a (clone HIT8a)	BioLegend	Cat# 300922, RRID: AB_1575072)
Brilliant Violet 510 mouse anti-human CD14 (clone M5E2)	BioLegend	Cat# 301842, RRID:AB_2561946
BV786 mouse anti-human CD16 (clone 3G8)	BD Biosciences	Cat# 563690, RRID:AB_2744299
BB700 mouse anti-human CCR2 (CD192) (clone 1D9)	BD Biosciences	Catalog No. 747847, RRID:AB_2861365
Brilliant Violet 421 mouse anti-human CD193 (CCR3) (clone 5E8)	BioLegend	Cat# 310714, RRID: AB_2561886
PE-CF594 Mouse Anti Human CD195 (CCR5) (clone 2D7/CCR5)	BD Biosciences	Cat# 562456, RRID: AB_11154599
Brilliant Violet 711 mouse anti-human CD196 (CCR6) (clone G034E3)	BioLegend	Cat# 353436, RRID:AB_2629608
LIVE/DEAD Fixable Near-IR Stain	Invitrogen	Cat# L10119
CellROX™ Deep Red Flow Cytometry Assay Kit	Invitrogen	Cat# C10491
**Biological Samples**		
Human plasma samples	Karolinska University Hospital, Huddinge, Sweden	
Human PBMC samples	Karolinska University Hospital, Huddinge, Sweden	
**Critical Commercial Assays**		
Proximity Extension Assay (PEA) Immuno-Oncology Panel	Olink Bioscience, RRID:SCR_003899	Cat# 95310
Human C-Reactive Protein/CRP Quantikine ELISA Kit	R&D Systems	Cat# DCRP00
Human CCL20/MIP-3 alpha Quantikine ELISA Kit	R&D Systems	Cat# DM3A00
Neopterin ELISA	TECAN	Cat# RE59321
**Deposited Data**		
Metabolomics	This paper	Supplementary (Table S3)
Proteomics	Zhang et al, 2018; This paper	
Flow cytometry	This paper	Flow Repository ID: FR-FCM-Z2T3
Code	This paper	https://github.com/neogilab/METABO-EC
**Software and Algorithms**		
GraphPad Prism v8		https://www.graphpad.com
R	R Core Team 2020	https://www.r-project.org/
ggplot2 R package	Wickham et al, 2016	https://CRAN.R-project.org/package=ggplot2
Ropls R package	Thevenot et al, 2015	https://bioconductor.org/packages/ropls/
gplots R package	Warnes et al, 2005	https://CRAN.R-project.org/package=gplots
Stats R package	R Core Team 2020	https://www.r-project.org/
Cytoscape v3.6.1	Shannon et al, 2003	https://cytoscape.org/
FlowJo™ v10		https://www.flowjo.com/

Spice	Roederer et al, 2011	https://niaid.github.io/spice/
Adobe Illustrator v24		https://www.adobe.com

SUPPLEMENTARY REFERENCE

Babu, H., Sperk, M., Ambikan, A.T., Rachel, G., Viswanathan, V.K., Tripathy, S.P., Nowak, P., Hanna, L.E., and Neogi, U. (2019). Plasma Metabolic Signature and Abnormalities in HIV-Infected Individuals on Long-Term Successful Antiretroviral Therapy. Metabolites *9*.

Bar, N., Korem, T., Weissbrod, O., Zeevi, D., Rothschild, D., Leviatan, S., Kosower, N., Lotan-Pompan, M., Weinberger, A., Le Roy, C.I.*, et al.* (2020). A reference map of potential determinants for the human serum metabolome. Nature *588*, 135-140.

RCoreTeam (2020). R: A language and environment for statistical computing. R Foundation for Statistical Computing (VIenna, Austria).

Roederer, M., Nozzi, J.L., and Nason, M.C. (2011). SPICE: exploration and analysis of post-cytometric complex multivariate datasets. Cytometry A *79*, 167-174.

Shannon, P., Markiel, A., Ozier, O., Baliga, N.S., Wang, J.T., Ramage, D., Amin, N., Schwikowski, B., and Ideker, T. (2003). Cytoscape: a software environment for integrated models of biomolecular interaction networks. Genome research *13*, 2498-2504.

Thévenot, E.A., Roux, A., Xu, Y., Ezan, E., and Junot, C. (2015). Analysis of the Human Adult Urinary Metabolome Variations with Age, Body Mass Index, and Gender by Implementing a Comprehensive Workflow for Univariate and OPLS Statistical Analyses. Journal of Proteome Research *14*, 3322-3335.

Warnes, G., Bolker, B., Bonebakker, L., Gentleman, R., Huber, W., Liaw, A., Lumley, T., Mächler, M., Magnusson, A., and Möller, S. (2005). gplots: Various R programming tools for plotting data, Vol 2.

Wickham, H. (2016). ggplot2. Elegant Graphics for Data Analysis (New York: Springer International Publishing).

Zhang, W., Ambikan, A.T., Sperk, M., van Domselaar, R., Nowak, P., Noyan, K., Russom, A., Sonnerborg, A., and Neogi, U. (2018). Transcriptomics and Targeted Proteomics Analysis to Gain Insights Into the Immune-control Mechanisms of HIV-1 Infected Elite Controllers. EBioMedicine *27*, 40-50.
